# Serum Metabolomic Profiles for Breast Cancer Diagnosis, Grading and Staging by Gas Chromatography-Mass Spectrometry

**DOI:** 10.1038/s41598-017-01924-9

**Published:** 2017-05-11

**Authors:** Naila Irum Hadi, Qamar Jamal, Ayesha Iqbal, Fouzia Shaikh, Saleem Somroo, Syed Ghulam Musharraf

**Affiliations:** 1 Department of Pathology, Ziauddin University, Clifton, Karachi, 75600 Pakistan; 2 Dr. Panjwani Center for Molecular Medicine and Drug Research, International Center for Chemical and Biological Sciences, University of Karachi, Karachi, 75270 Pakistan; 3 Breast Clinic, Surgical Ward 2, Jinnah Postgraduate Medical Center (JPMC), Karachi, 75510 Pakistan; 4 H.E.J. Research Institute of Chemistry, International, Center for Chemical and Biological Sciences, University of Karachi, Karachi, 75270 Pakistan

## Abstract

Detection of metabolic signature for breast cancer (BC) has the potential to improve patient prognosis. This study identified potentially significant metabolites differentiating between breast cancer patients and healthy controls to help in diagnosis, grading, staging and determination of neoadjuvant status. Serum was collected from 152 pre-operative breast cancer (BC) patients and 155 healthy controls in this case-controlled study. Gas chromatography-mass spectrometry (GC-MS) was used to obtain metabolic profiles followed by chemometric analysis with the identification of significantly differentiated metabolites including 7 for diagnosis, 18 for grading, 23 for staging, 15 for stage III subcategory and 10 for neoadjuvant status (*p*-value < 0.05). Partial Least Square Discriminant Analysis (PLS-DA) model revealed a distinct separation between healthy controls and BC patients with a sensitivity of 96% and specificity of 100% on external validation. Models for grading, staging and neoadjuvant status were built with Decision Tree Algorithm with predictive accuracy of 71.5%, 71.3% and 79.8% respectively. Pathway analysis revealed increased glycolysis, lipogenesis, and production of volatile organic metabolites indicating the metabolic alterations in breast cancer.

## Introduction

Breast cancer (BC), ranked as the fifth amongst all cancers, is the commonest type of cancer in women both in developed and developing countries, with approximately 1.67 million new cases reported in 2012^1^. Five-year survival rates can be improved to more than 90% by diagnosis at an early stage, in contrast to 15% in women diagnosed at an advanced stage^2^. Mammographic diagnosis of breast cancer contributes to treatment success, which can further be improved with the use of blood-based biomarkers, especially for younger women as mammography is less sensitive in this group^3^.

Metabolomics provides identification of altered metabolites (mol. wt <1500 kDa) in cancer tissues and biofluids^4^. Mass spectrometry (MS) and Nuclear magnetic resonance (NMR) spectroscopy are commonly used analytical techniques for metabobolmics, along with multivariate statistical analysis^5^. Studies conducted on breast cancer highlight the importance of metabolomics not only for diagnosis, monitoring response to treatment, but also for finding a relationship between clinicopathological characteristics of breast cancer and different metabolites in blood, urine and tissue specimens^6–11^. There are few reports with characteristic up fold/down fold changes of metabolites in breast cancer patients by GC-EI-MS. In a study carried out on invasive breast cancer by GC-TOF MS, the metabolite ratio of cytidine-5-monophosphate to pentadeconic acid differentiated between cancer tissue and normal breast tissue with high sensitivity and specificity^11^. BC and normal breast tissue were also differentiated by distinctly elevated choline peak intensities on High-Resolution Magic Angle Spinning (HRMAS) analysis^10^. Similarly, Brockmoller and colleagues found altered lipid metabolites, with elevated levels of glycerol-3-phosphate and phospholipids in BC tissues^12^. In studies carried out on exhaled breath, increased levels of volatile organic metabolites (VOM) were detected in BC patients by Gas chromatography-mass spectrometry (GC-MS)^13, 14^. Early detection of recurrent BC by a combination of techniques (NMR and GC x GC – MS) yielded a distinct serum metabolic profile^15^.

Prognosis for women with BC depends not only on an early diagnosis, but also the grade and stage of tumor (extent of spread) at the time of presentation^16^. However, there are few reports on histologic grading of BC based on metabolic profiling^10, 17–19^. In the present study, serum metabolomic analysis of a large number of breast cancer patients (n = 152) and healthy controls (n = 155) was carried out by GC-MS with the aim to identify distinct metabolic profiles for the prediction of BC diagnosis, grading, staging and neoadjuvant status, to improve the management of BC significantly and highlight the potential of these metabolic signatures as prognostic factors.

## Material and Methods

### Patient Selection

The present study was conducted in accordance with declaration of Helsinki and was approved by the Ethics Review Committee of Ziauddin University (0420612NIPATH). Written informed consent was obtained from all the participants of this study including controls and patients. This was a prospective study which included 152 cases of invasive breast cancer (age = 46.69 ± 10.53 years, age range 22 to 76 years) and mean body mass index (BMI) of 20.56 ± 1.32 kg/m^2^ with following inclusion criteria: histologic diagnosis by a trucut biopsy, availability of both blood and tissue samples and patient fasting for a minimum of 6–8 hours prior to enrollment for blood sample. Exclusion criteria included malignant mesenchymal tumors of the breast and history of other cancers. Patients received no chemotherapy, hormonal therapy or radiotherapy prior to surgery. A group of 31 breast cancer patients (age = 45.55 ± 9.31 years, age range 25 years to 70 years) with history of pre-operative neoadjuvant chemotherapy comprising of TAC regimen (Taxotere 75 mg/m^2^ + doxorubicin 50 mg/m^2^ + cyclophosphamide 500 mg/m^2^ IV given once every 3 weeks with a total of 6 cycles) were also included. Detailed patient characteristics are given in Table 1. One hundred and fifty-five age-matched healthy females (age = 44.56 ± 10.85 years, age range 20 years to 73 years) with a mean body mass index (BMI) of 22.68 ± 1.38 kg/m^2^ were also included in this study. Mammography and ultrasonography was done to exclude any breast pathology. In order to reduce variation in data, both the healthy control and breast cancer patients were matched for ethnicity as they belonged to diverse ethnic groups namely: Muhajirs (Urdu speaking), Punjabi, Pathans, Baluchis, Sindhis and Others. The latter category of ‘Others’ included minor ethnic groups like Seraiki, Gujarati, Hindku etc.

**Table 1:** Characteristics of Breast Cancer Patients.

		Frequency (Percentage)	
Parameter		Pre-operative (n = 152)	Neoadjuvant (n-31)
Diagnosis	IDC	147 (96.7)	28 (90.32)
	ILC	5 (3.3)	3 (9.68)
Side/Laterality	Right	67 (44.07)	18 (58.06)
	Left	71 (46.71)	12 (38.71)
	Bilateral	4 (2.63)	1 (3.23)
	Unknown	10 (6.57)	0 (00)
Age Group (years)	Premenopausal	74 (48.6)	17 (54.84)
	Post-menopausal	78 (50.6)	14 (45.16)
Pathologic Stage (TNM)	I	4 (2.6)	2 (6.45)
	II	35 (23.02)	14 (45.16)
	III	67 (44.07)	13 (41.94)
	IV	14 (9.21)	2 (6.45)
	Unknown	32 (21.05)	0 (00)
Histologic Grade (Modified B-R)	I	8 (5.3)	1 (3.23)
	II	70 (46.1)	15 (48.39)
	III	61 (40.1)	13 (41.94)
	Unknown	13 (8.6)	2 (6.45)

### Blood Sample Collection

Blood samples of female breast cancer patients and healthy controls were collected from Jinnah Postgraduate Medical Center (JPMC), Karachi and Ziauddin University Hospital, Karachi. About 4 mL of blood was drawn from fasting pre-operative breast cancer patients and collected in 5 mL gel-based BD^®^ vacutainer plus SST™ tubes (BD Franklin Lakes NJ, USA, REF: 367986). Serum was separated by centrifugation at 4000 rpm for 10 minutes at 4 °C with 200 µL serum stored in 1.5 mL aliquots (Eppendorf^®^ safe-lock microcentrifuge tubes purchased from Sigma-Aldrich, REF: T9661). In this way each sample was stored in multiple aliquots (about 5–6) and immediately shifted to −80 °C freezer.

### Tissue Sample Collection and Processing

Breast cancer tissue samples obtained through surgery were fixed in 10% buffered formalin and sent to Ziauddin Hospital Histopathology Laboratory for detailed morphological and immunohistochemical examination for ER, PR and HER2/neu. ER and PR quality control and reporting was performed using ASCO/CAP guidelines^20, 21^. For HER2 expression patients with scores of ‘0’, ‘1+’ and ‘2+’ were recorded as negative while patients with scores of ‘3+’ were recorded as positive^22^.

Tumors were graded by the Nottingham Histologic Score (also referred to as Modified Scarff-Bloom-Richardson system), which combines nuclear grade, tubule formation, and mitotic activity (1/10 HPF) to classify invasive carcinomas into three grades (I, II and III)^23^. ASCO/CAP guidelines were followed for pathologic staging of breast cancer from stage 0 to stage IV by TNM staging system (pTNM)^24^.

### Reagents and Solvents

Analytical grade solvents were used for GC-MS analysis. Reagents and solvents included methanol, hexane (Tediaway, Fairfield, USA), myristic-d_27_ acid, *N*-Methyl-*N*- (trimethylsilyl) trifluoroacetamide (MSTFA), methoxylamine hydrochloric (Acros Organic, New Jersey, USA), Pyridine (Lab-Scan, Bangkok, Thailand) and deionized water (Milli-Q) (Millipore, Billerica, MA, USA).

### Sample Preparation for GC-MS Analysis

Sample preparation protocol reported previously was used for this study with some minor modifications^25^. Sample processing in a 96 well plate (Strata C18-E, 55 µm pore size, 70 Å particle, 100 mg sorbent/1 mL Phenomenex, USA) included the following steps. First stock solution with a concentration of 1 mg/mL was prepared for myristic-d_27_ acid, which was used as an internal standard (IS). Then 100 µL serum of each case was mixed with 800 µL methanol followed by addition of 20 µL internal standard (IS). This mixture was vortexed and left on ice for 30 minutes. Then the mixture was centrifuged (Eppendorf Centrifuge 5804 C/R) for 20 minutes at 3,500 rpm to remove the precipitated protein. Clear supernatant was poured into the well plate and drawn through the solid phase under vacuum (AHC-7502, Phenomennex, USA). Before extraction, solid phase was activated with 600 µL methanol and 600 µL deionized water (Milli Q). After sample loading, the solid phase was washed with 300 µL of water and eluted with 600 µL methanol with eluates collected in 96 well collection plates. Finally the eluate was evaporated under nitrogen (N_2_) at room temperature. Samples were dried in Eppendorf™ Concentrator (5301) for approximately 4–5 hours (or till they were completely dry) and stored at 4 °C till further analysis.

Sample derivatization involved addition of 50 µL methoxylamine hydrochloride in 15 µg/µL pyridine followed by mixing at 600 rpm (Eppendorf™ Thermomixer) for 2 hours at 35 °C. This was followed by addition of 50 µL MSTFA (with 1% trimethylchlorosilane) mixing for 60 minutes at 70 °C, with formation of trimethylsilyl derivatives. Then sample was centrifuged at 14,000 rpm for 10 minutes. The clear supernatant was shifted quickly to inserters in the labeled GC vials for GC-MS analysis, leaving the precipitate at the bottom.

### GC-MS Analysis

GC-MS analysis was carried out on 7890A GC (Agilent Technologies, USA) fitted with a GC sampler 120 (PAL LHX-AG12 – Agilent Technologies) autosampler and coupled to Agilent 7000 Triple Quad system (Agilent Technologies, USA). A fused-silica capillary GC column, HP-5MS 30 m × 0.25 mm ID (Agilent J &W Scientific, Folsom, CA, USA), chemically bonded with a 95% dimethylpolysiloxane 5% diphenyl cross-linked stationary phase (0.25 mm film thickness) was used as previously reported^26^. The serum sample was inserted in the splitless mode using helium as carrier gas. Initially the oven temperature was fixed at 40 °C, then increased to 300 °C at a rate of 10 °C per minute. After maintaining the temperature at 300 °C for nine minutes, it was further increased to 305 °C for one minute. Retention time was locked to the internal standard at 15.168 minutes^27^. Electron impact ionization (EI) was used as an ionization source for the GC/MS analysis at 70 eV. GC-MS analysis was performed according to the GC parameters also described previously^25^. Data acquisition was done in full scan mode from 50–650 *m*/*z* in 0.5 seconds scan time. Hexane (as blank) was run between samples to remove contamination. Mass calibration was done with perfluorotributylamine (PFTBA). A quality control (QC) sample was run after every six samples of each batch of 23 samples during GC-MS analysis.

### GC-MS Data Preprocessing and Statistical Analysis

Agilent Mass Hunter Qualitative Analysis software (version B.04.00) was used for data processing. Peak integration and deconvolution parameters have been previously reported^25^. Mass spectra of the peaks were compared with NIST mass spectral (Wiley registry NIST 11) and Fiehn RTL libraries for presumptive identification of metabolites with a ≥70% similarity index. The GC-MS spectra were uploaded on Mass Profiler Professional (MPP) software 12.5. Filtering of the data involved using all available data with minimum absolute abundance of 5,000 counts. Match factor 0.3, retention time tolerance 0.05 and delta MZ (low resolution) 0.2 were set as alignment parameters. Data normalization was carried out which transforms data to log_2_ scale and then additional normalization was carried out by external scalar which assign a value to scale up or scale down the provided samples. This normalization process subtracts the external scalar *S*_*l*_ (for the *l* ^*th*^ sample) to each abundance value *m*_*jl*_. The normalized abundance, *m*_*jl*_ = log_2_ *m*_*jl*_ − log_2_ *S*_*l*_. Z transform was selected as baselining option treating all the compounds equally irrespective of their intensity. A total of 424 compounds were detected in the entire samples (list is provided in Supplementary Dataset 1) and were filtered by frequency (i.e. compounds appearing in more than 50% of samples in at least one group of samples) and p-value < 0.05. Statistical significance analysis was done using student T-test for healthy versus breast cancer patients of fold change (FC) 1.5, while one way ANOVA was used for healthy versus grades of breast cancer (FC 2), healthy versus stages of breast cancer (FC 2) and healthy versus neoadjuvant group of breast cancer (FC 1.5). Sub-categories of stage II (A, B and C) were also analyzed using one-way ANOVA (FC 2). Tukey’s Honest Significant Difference (HSD) post-hoc test was then applied to identify which entities were responsible for significant differences amongst the different groups i.e. healthy versus grades, healthy versus stages and sub-categories of stage III and healthy versus neoadjuvant group. Hierarchical clustering was performed by applying Canberra distance metric and complete linkage amalgamation algorithm. A PLS-DA model was built for healthy versus breast cancer using auto scaling, N fold validation type, three number of folds and ten number of repeats. Randomly selected samples (n = 100) were validated through external validation. Prediction models were also built for healthy versus grades, stages and neoadjuvant status. A Decision Tree model was built for healthy versus grades using minimum error as pruning method, N fold validation, two number of folds, and twenty number of repeats with 0.3 as attribute factor at nodes, while model for staging was built using Leave-one-out cross-validation (LOOCV). Decision Tree models for healthy versus stage III subcategories and neoadjuvant status were also built using pessimistic error as pruning method, N fold validation, two number of folds, and thirty number of repeats with attribute factor of one for stage III and 1.5 for neodjuvant group. Sensitivity, specificity and predictive accuracy of the constructed model were also measured.

### Results and Discussion

Metabolite profiling of a total of 307 serum samples from healthy females (155) and breast cancer patients (152) was performed by using GC-EI-MS. 2D-C18 sample preparation method was used for the enrichment of metabolites^26^. Before in-depth analysis, the overall picture of 424 metabolites in the healthy controls and BC patients was obtained through a Principal Component Analysis (PCA) score plot (Fig. S1 of supplementary information), which showed variation in the two groups with formation of distinct clusters of both groups with overlapping of some samples due to no filtration of data at this stage. Significance testing, fold change and hierarchical clustering were performed by classifying the data on the basis of four parameters i.e. health status, grade, stage and neoadjuvant status.

### Diagnosis of Breast Cancer

A total of 152 BC samples were compared with 155 healthy controls. Seven metabolites were found to be significantly different among healthy controls and breast cancer patients using the student’s T test and a level of probability of 0.05 and fold change >1.5 (Table S1 of supplementary material). Before detailed analysis we grouped healthy and BC patients on the basis of age. Principal Component Analysis (PCA) showed no variation between the different groups of healthy controls (Fig. S2 of supplementary material) as well as BC patients (Fig. S3 of supplementary material) indicating that age has no effect on metabolite pattern, while the separation of samples is occurring due to heath status.

**Figure 1:**
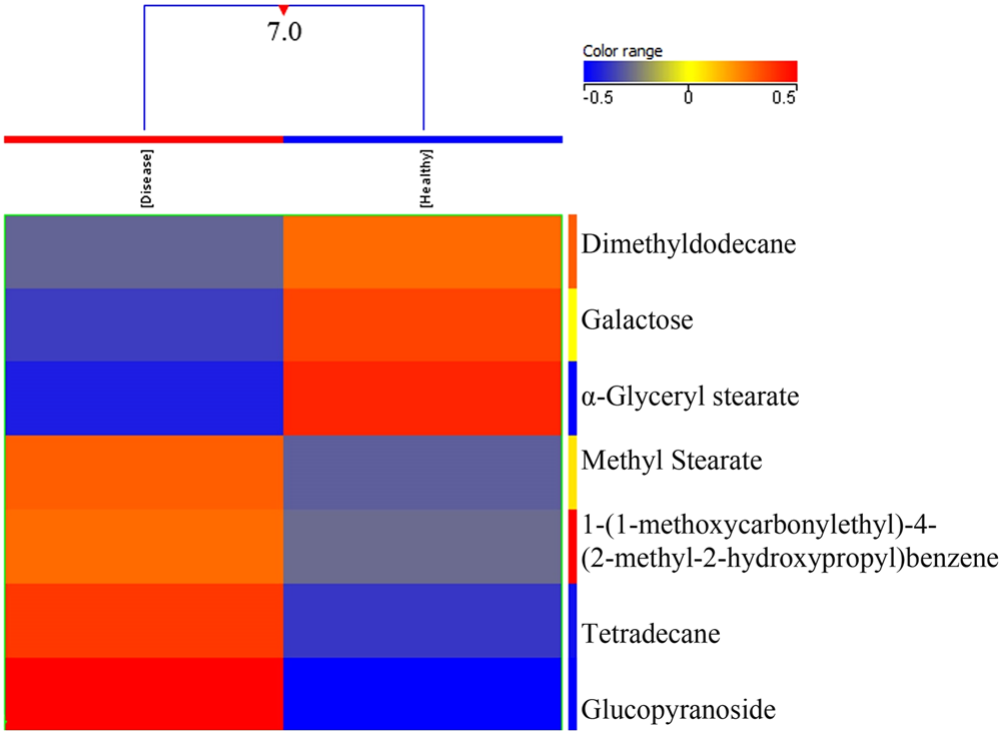
Dendogram showing comparison of healthy controls and Breast Cancer patients using normalized intensities of seven significant metabolites (p < 0.05). The dendogram was constructed by applying a hierarchical clustering algorithm (Canberra, absolute distance metric, complete linkage).

Using normalized intensities of seven metabolites significantly different between healthy controls and breast cancer patients, a dendogram was produced through Hierarchical cluster analysis (HCA) (Fig. 1) which involves organizing metabolites into clusters on the basis of their profile similarity. HCA approach clustered the two groups into healthy and breast cancer patients with a high level of dissimilarity. A heat map was also generated using all samples with normalized intensities of seven significant metabolites (Fig. S4 of supplementary material).

The average coefficient of variation (CV) of the differential metabolites in QC samples was also calculated in order to check and monitor day-to-day and batch-to-batch variation in performance. The CV of each metabolite was as follows: tetradecane (−1.14783E-05), alpha-D-glucopyranoside (−4.47826E-06), methyl stearate (−6.08696E-06), dodecane (−7.6087E-06), 1-4-benzene (1.44348E-05), D-galactose (−2.6087E-06) and octadecanoic acid (1.02609E-05).

### Grades of Breast Cancer

A total of 139 BC samples (with known histological grades) were compared with 155 healthy controls. Eighteen metabolites were found significantly differentiated among healthy controls and three grades of breast cancer. Fifteen metabolites were putatively identified (Table S2 of supplementary material). The EI/MS spectra of three unidentified compounds are shown in supplementary information (Fig. S5). Different grades of BC were adequately stratified (grade I to III) showing a significant fold change when compared with healthy controls. An interesting observation was that with all the metabolites higher FC was seen in the grade I and III BC patients. Tukey’s HSD post hoc test was applied to find significantly expressed metabolites among healthy controls and three grades of breast cancer. The differential expression of metabolites revealed a distinct pattern, with most differentiation seen between healthy controls and grade I BC patients (18 metabolites expressed) followed by grade III and grade II BC patients (13 and 10 metabolites expressed respectively) (Table S3 of supplementary material).

**Figure 2:**
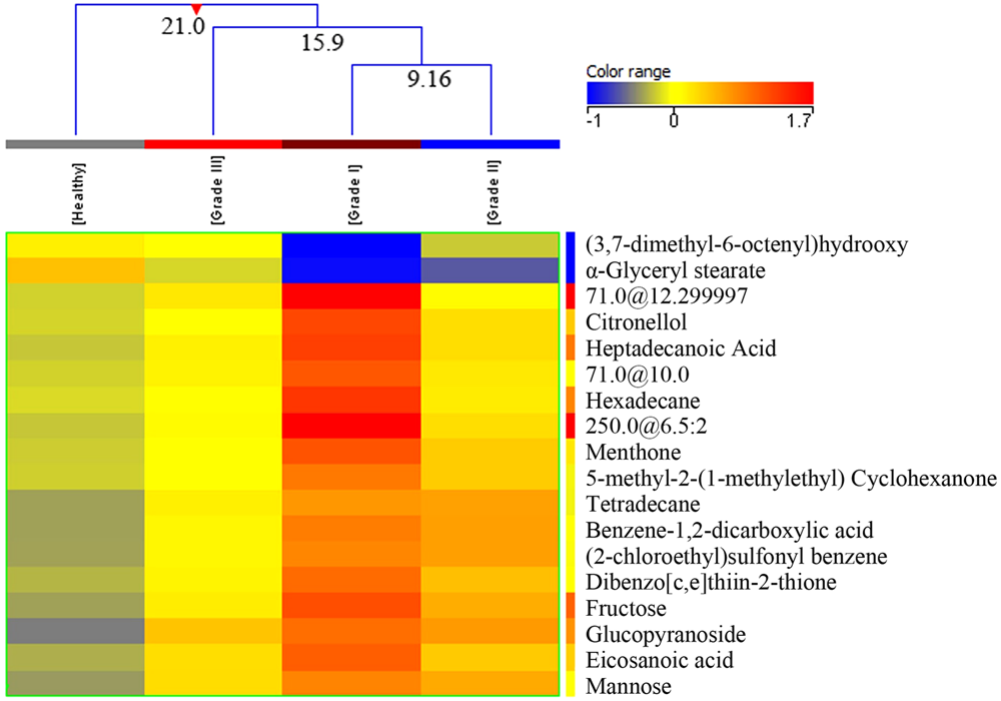
Dendogram showing comparison of four groups of samples i.e. healthy controls, grade. I, II and III of Breast Cancer patients using normalized intensities of eighteen significant metabolites (p < 0.05). The dendogram was constructed by applying a hierarchical clustering algorithm (Canberra, absolute distance metric, complete linkage). Compounds that have been identified have their name mentioned while unidentified compounds are recognized by their retention time.

Hierarchical clustering was done using normalized intensities of eighteen compounds which were significantly different between healthy controls and grades (I, II and III) of breast cancer patients. The four groups were clustered into three classes (Fig. 2). Grades I and II were clustered together in class I with a dissimilarity of 9.16, while grade I, II and III were clustered together in class II with a dissimilarity level of 15.9. All the four groups (healthy controls, grade I, grade II and grade III BC patients) were clustered in class III revealing a high dissimilarity level of 21.0 indicating that healthy controls were most dissimilar from the three groups of breast cancer patients (Fig. 2). A heat map was also produced using all samples (healthy as well as BC) with normalized intensities of eighteen significant metabolites (Fig. S6 of supplementary material).

Clinical characteristics of grade II and III tumors are comparable and hence smaller differences are reported in the chemical properties as well. Cheng *et al*.^28^ studied BC tissue by HR MAS MRS and reported differences in metabolite levels with different histopathological grades of BC for the first time. They reported a subsequent increase in PC/Cho ratio from IDC grade II to IDC grade III. In contrast, Sitter *et al*.^9^ reported a relative decrease in this ratio from grade II to III indicating an increase in choline. Beckonert *et al*.^18^ analyzed BC tissue from 49 patients and compared it with normal breast tissue from 39 healthy women using 1H-NMR spectroscopy. They also reported findings similar to Sitter *et al*.^9^, with higher levels of PC and taurine in grade II and III IDC compared with grade I. Previous studies on grading of breast cancer have reported comparable results in various studies carried out on cancer tissue by NMR spectroscopy^9, 10, 17, 18, 29^. They grouped grade I and II BC patients as “low-grade” and grade III as “high-grade” cancers with distinct metabolic differences between the two groups.

Our study employed GC-MS for analysis of a larger cohort of BC patients (n = 152) and found significantly expressed metabolites differentiating between the three grades. These differences were highlighted in the hierarchical clustering and Tukey’s post Hoc test. Till recently grade II and III tumors were given more or less similar adjuvant therapy but metabolic differences between the two grades might be helpful in devising different treatment regimens in the future.

### Stages of Breast Cancer

**Figure 3:**
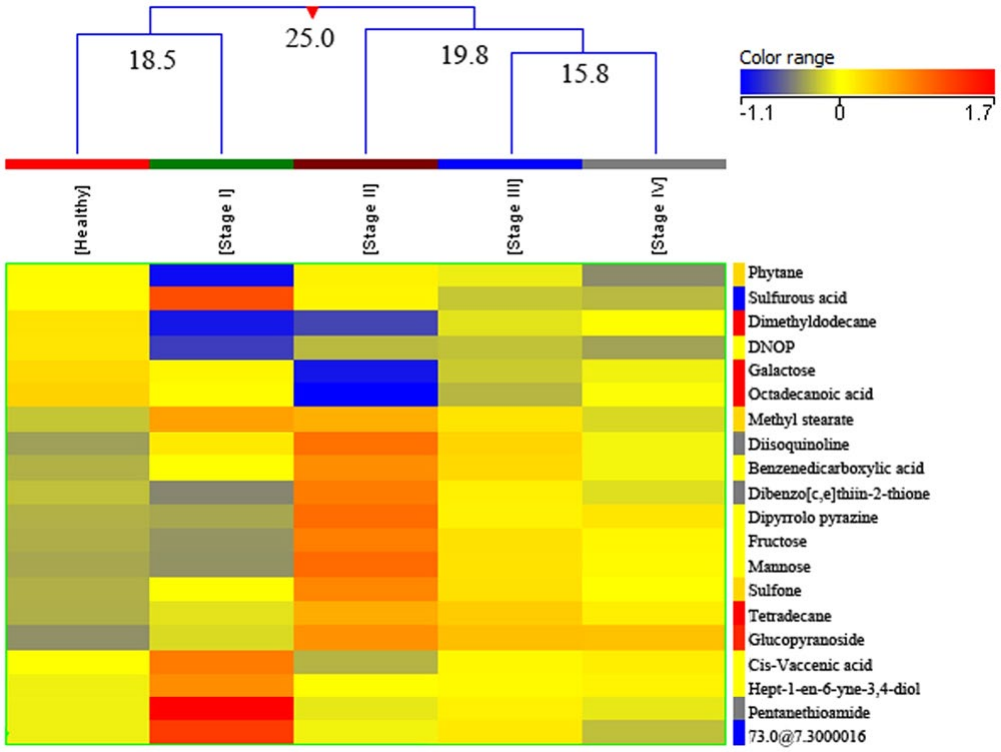
Dendogram showing comparison of five groups i.e. healthy controls, stage I, stage II, stage III and stage IV of Breast Cancer patients using normalized intensities of twenty significant metabolites (p < 0.05). The dendogram was constructed by applying a hierarchical clustering algorithm (Canberra, absolute distance metric, complete linkage). Compounds that have been identified have their name mentioned while one of the unidentified compound is recognized by its retention time. (DNOP - (1,2 Benzenedicarboxylic acid, bis (2-ethylhexyl) ester).

A total of 120 BC samples (with known pathological stages) were compared with 155 healthy controls. Before analysis of these two groups the PCA score plot was obtained for all metabolites in healthy controls and different stages of BC showing clusters of healthy and BC with little variation between different stages of BC (Fig. S7 of supplementary information). Twenty metabolites distinguished between healthy controls and four stages of breast cancer patients among which nineteen metabolites were putatively identified (Tables S4a and S4b of supplementary material). The EI/MS spectrum of the unidentified compound is shown in supplementary information (Fig. S8). Different stages of BC were adequately stratified (TNM staging: I to IV) showing a significant fold change when compared with healthy controls. An interesting observation was that with all the metabolites the greatest FC was seen in stage II BC patients. Tukey’s HSD post hoc test showed that the stage I BC patients differed significantly from healthy controls as well as stage II patients as compared to stage III BC patients. Although stage IV revealed significant differentiation from stage I and II, a small difference was observed between stage III and IV showing a close resemblance between these two stages (Table S5 of supplementary material). The five groups were clustered by hierarchical clustering into four classes (Fig. 3). All the five groups (healthy controls, stage I, stage II, stage III and stage IV BC patients) were clustered in class IV revealing a high dissimilarity level of 25.0, indicating that healthy controls were most dissimilar from the three groups of breast cancer patients (Fig. 3). A heat map was also produced using all samples (healthy as well as BC) with normalized intensities of twenty significant metabolites (Fig. S9 of supplementary material).

**Figure 4:**
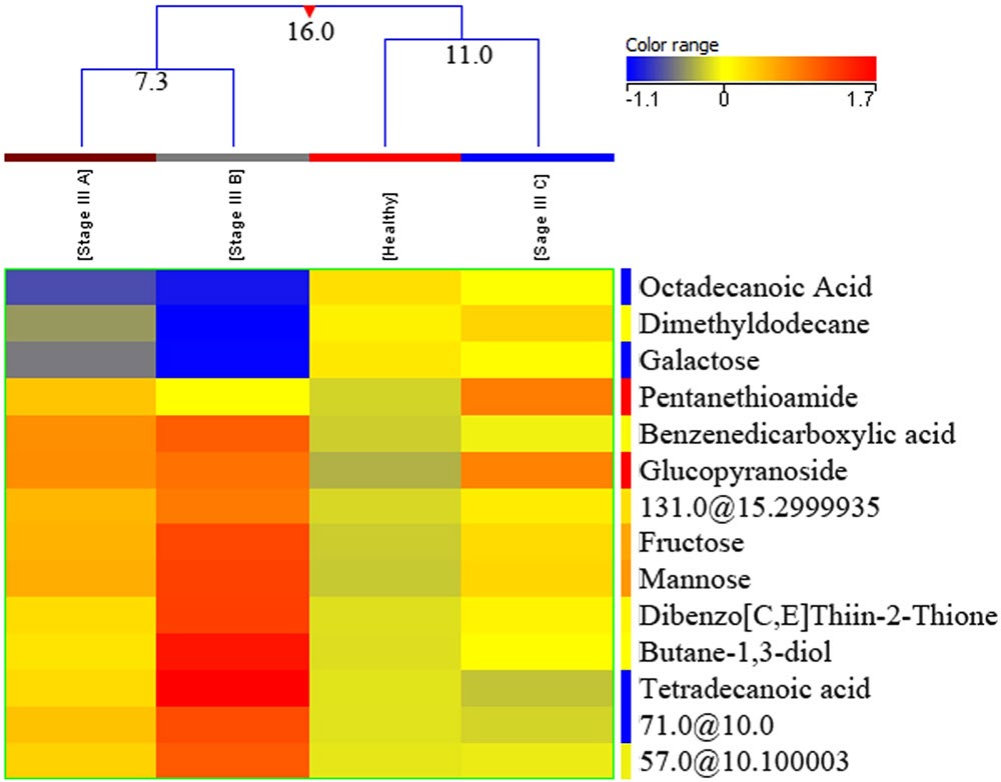
A dendogram showing comparison of four groups i.e. healthy controls, stage IIIA, stage IIIB and stage III C of Breast Cancer patients using normalized intensities of fourteen significant metabolites (p < 0.05). The dendogram was constructed by applying a hierarchical clustering algorithm (Canberra, absolute distance metric complete linkage). Compounds that have been identified have their names mentioned while three of the unidentified compounds are recognized by their retention time.

A total of 67 stage III BC samples were compared with 155 healthy controls to find out intra-stage variation in metabolite expression. Fourteen metabolites distinguished between healthy controls and three pathologic sub-categories of stage III (A, B, C) breast cancer of which, eleven metabolites were putatively identified (Table S6 of supplementary material). The EI/MS spectra of three unidentified compounds are shown in supplementary information (Fig. S10). An interesting observation was that with all the metabolites the greatest FC was seen in stage IIIB BC patients. Tukey’s HSD post hoc test was also applied on this group and its summary is shown in supplementary information (Table S7). A dendogram was produced dividing the four groups (healthy control, stages III A, III B and III C of BC patients) into three classes (Fig. 4). Stages III A and III B were clustered together in class I with a dissimilarity of 7.3. Healthy controls and stage III C were clustered together with a dissimilarity of 11.0 while all the four groups (healthy controls, stages III A, III B and III C of BC patients) clustered together in class III showed the highest level of dissimilarity (16.0) (Fig. 4). A heat map was also produced using all samples (healthy as well as BC) with normalized intensities of fourteen significant metabolites (Fig. S11 of supplementary material).

By finding metabolic differences between the four stages of BC and stage III subcategories (A-C), an attempt was made to highlight the sensitivity of this technique for studying both inter- as well as intra-stage variation in metabolite levels. These metabolic differences between stages might also be helpful in devising different treatment regimens in the future. We could not find any metabolomics study reporting significant metabolic differences with different stages of BC.

### BC Subtypes

On histology most of the tumors were invasive ductal carcinoma (IDC) (142; 93.4%) with only 7 cases (4.6%) of invasive lobular carcinoma (ILC), two cases (1.3%) of metaplastic breast cancer and one case (0.67%) of mucinous carcinoma. However majority of patients are invasive ductal carcinoma with lesser number of other histological types to draw any definite conclusion. Therefore we segregated these cases on the basis of ER, PR and HER2 into distinct BC subtypes. There were 77 (51.3%) ER positive, 61 (40.7%) PR positive, 47 (31.3%) Her2/neu positive, 25 (18.8%) triple-negative BC (TNBC), 27 (20.3%) triple-positive BC (TPBC) and 104 (78.8%) non-triple negative BC (nTNBC) patients. Significant metabolic differences (FC 1.5-2 at p < 0.05) were observed between healthy and ER expression (ER+ and ER−), PR expression (PR+ and PR−) and HER2 expression (HER2+ and HER2−). The metabolic differences can be appreciated in the dendograms for clustering of ER+/ER−, PR+/PR− and HER2+/HER2− given in supplementary information (Fig. S12a,b and c of supplementary information).

### Neoadjuvant BC Patients

**Figure 5:**
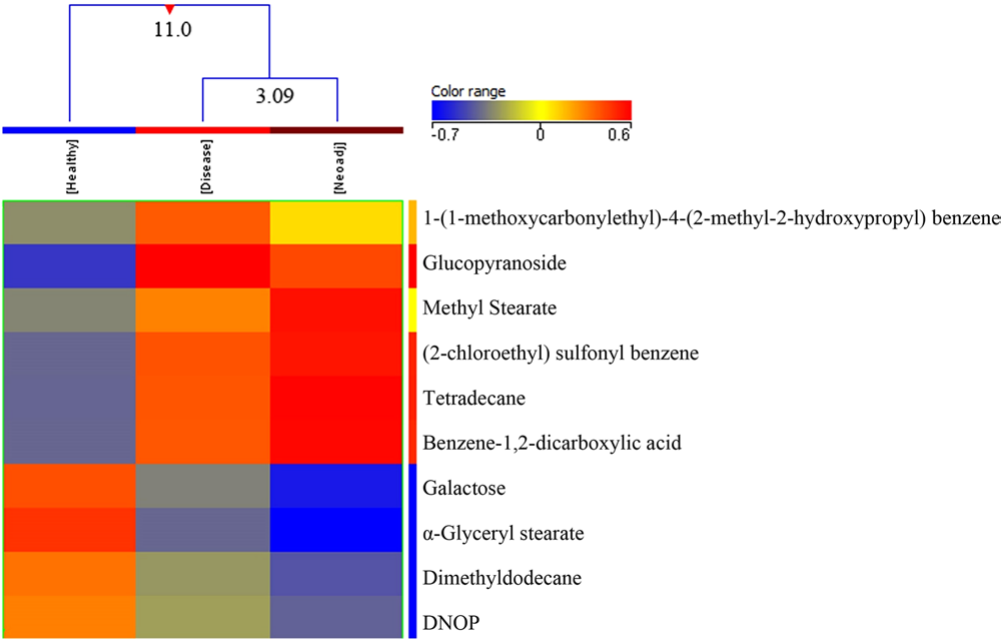
A dendogram showing comparison of three groups i.e. healthy controls, BC patients and Breast Cancer patients on neoadjuvant therapy using normalized intensities of ten significant metabolites (p < 0.05). The dendogram was constructed by applying a hierarchical clustering algorithm (Canberra, absolute distance metric, complete linkage). Compounds that have been identified have their names mentioned. (DNOP - 1,2 Benzenedicarboxylic acid, bis (2-ethylhexyl) ester).

A total of 31 neoadjuvant BC and 152 BC samples were compared with 155 healthy controls and ten metabolites were distinguished between these groups (Table S8 of supplementary material). The neoadjuvamt group of BC patients showed a significant fold change when compared with healthy controls. In the Tukey’s HSD post hoc test, 10 metabolites significantly differentiated healthy controls from breast cancer patients while 9 metabolites differentiated between healthy controls and neoadjuvant group, thus revealing a significant difference between these groups (Table S9). No significant metabolites were detected between neoadjuvant and BC patients indicating their diseased status. Normalized intensities of ten metabolites were used to divide the three groups (healthy control, breast cancer patients and BC patients receiving neoadjuvant therapy) into two classes (Fig. 5). In the dendogram, BC patients and the neoadjuvant group were clustered together in class I with a dissimilarity of 3.09, while the three groups clustered together in class II showed a higher dissimilarity level of 11.0 (Fig. 5). A heat map was also produced using all samples with normalized intensities of ten significant metabolites (Fig. S13 of supplementary material).

Women with locally advanced BC (LABC) or large tumors require neoadjuvant chemotherapy (NAC). Patients can be spared unnecessary use and side effects of drugs by predicting their response to NAC before or at the start of treatment^30^. Heterogeneity of BC leads to variable response to treatment.

### Class Prediction Model and Test

**Figure 6:**
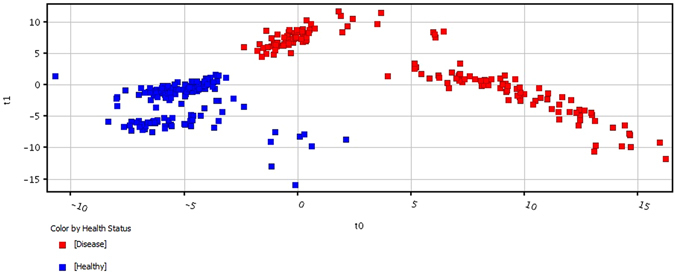
PLS-DA scores scatter plots discriminating among healthy controls and breast cancer patients based on seven significantly differentiated metabolite profiling data. The blue squares indicate healthy controls (n = 155) while the red squares denote Breast Cancer patients (n = 152) respectively. (PLS-DA: Partial Least Squares-Discriminant Analysis).

A prediction model of healthy versus BC was built using seven statistically significant metabolites. Approximately 70% samples (n = 215) were used for model generation and 30% samples (n = 100) for testing the validity of the model. Samples were classified into discrete classes by Partial Least Square Discriminant Analysis (PLSDA). Two parts of the input data were randomly assigned to the training set and remaining into the testing set. Auto-scaling was applied which involves subtracting the variable mean from each variable (the data column) and dividing each by its standard deviation. This process was repeated ten times each time using a different part for testing thus using each row once in training and testing generating Confusion Matrix, which gives accuracy of prediction of each class (Table 2). Plots obtained by PLSD-A scores are shown in Fig. 6 displaying a clear separation trend between the healthy controls and breast cancer patients. However within the healthy as well as BC samples further smaller clusters can be seen. In healthy controls the major exclusion criteria was absence of BC, so further clustering could be due to presence of diseases like diabetes mellitus, hypertension etc. in some of the tested individuals. BC is a heterogeneous disease with distinctive biological subtypes and variable characteristics like lymph node involvement and distant metastasis. We assume that this could be the reason for clustering within the BC group. Sensitivity of the constructed model was calculated from the proportion of cancer samples which were correctly predicted (true positives), while specificity was determined from the proportion of control samples which were correctly predicted (true negatives). Sensitivity was found to be 100% and specificity was 97.3%, respectively, while the overall accuracy of the model was 98.6% (Table 2). The predictive capacity (i.e. sensitivity and specificity) of the model was measured by external validation using an independent or blind test set of 100 serum samples consisting of 50 samples each from healthy controls and breast cancer patients. External validation correctly predicted the presence of BC in 48 out of 50 patients and healthy controls in 50 out of 50 controls resulting in a sensitivity of 96% and specificity of 100%. Sample prediction reports are shown in Fig. S14 of supplementary information. To obtain the sensitivity and specificity of our prediction model we also built the receiver operating characteristic (ROC) plot. The ROC curve of our patients was found sufficiently good as the curve follows the left-hand border and the top border of the ROC space hence validating the accuracy of model (Fig. S15 of supplementary information). Also the area under the curve (AUC) which measures discrimination or is the ability of the test to correctly classify those with and without the disease was found to be 0.99 for both healthy and breast cancer patients. Although model needs to be validated on large number of samples.

**Table 2:** Summary report of PLS-DA Model generated on MPPfrom healthy controls (n = 100) and breast cancer patients (n = 105).

	Predicted Healthy	Predicted Breast cancer	Accuracy (%)
True Healthy	107	3	97.273
True Breast cancer	0	105	100.00
Overall Accuracy			98.605

PLS-DA – Partial Least Square Discriminant Analysis, MPP – Mass Profiler Professional.

Classification of healthy controls and cancer cases in our study, based on serum metabolomics analysis by GC-MS showed high specificity and sensitivity compared with previously reported figures. Relative intensities of metabolites showed significant differences between BC and normal samples. These findings are consistent with altered metabolism reported by others. There have only been few studies for comparison as those published have used either different analytical techniques or only selective clinicopathological parameters. For example, most of the researches on BC diagnosis employed different NMR techniques^8, 9, 18^, with few using GC-TOF-MS^11, 31^, or GC-MS^14, 32, 33^.

Sitter *et al*.^9^ attempted to diagnose BC on tissue biopsies with a sensitivity of 82% and specificity of 100%, while Slupsky *et al*.^8^ used NMR spectroscopy on urine samples to diagnose BC with a sensitivity of 100% and specificity of 93%. Budczies *et al*.^11^ also predicted BC with high accuracy (97% sensitivity and 93.9% specificity). Phillips *et al*.^33^ reported a high sensitivity of 94.1% with a comparatively lower specificity of 73.8%. Similarly another group of researchers using GC-MS reported figures of 78.5% and 88.3% as sensitivity and specificity, respectively for the training set, and 75.3% and 84.8% as sensitivity and specificity, respectively for the test set respectively^14^. Our results were in agreement with Slupsky *et al*.^8^ and Budczies *et al*.^11^, both showing high sensitivity and specificity for diagnosis of BC from normal healthy controls, but the former employs NMR and the latter GC-TOF-MS as analytical techniques. Moreover, the three studies also differed in the type of biological sample used for analysis i.e. serum, urine and breast tissue, respectively. The studies carried out on GC-MS differed from our study in two aspects; they reported lower sensitivity and specificity as compared to our study and breath was used as a sample in both these studies.

Prediction models were also built for grades, stages, and neoadjuvant status but external validation was not possible due to small number of cases in each subgroup and low sensitivity of the generated models. The Decision Tree algorithm was more robust for predicting these entities. Decision tree algorithm arranged as a tree uses a sequence of if-then-otherwise decisions. As the sample follows the appropriate path down the tree it gets classified. The predictive accuracy of the model for grading, staging and stage III subcategories was 71.5%, 71.3% and 78.3% respectively. Of the neoadjuvant BC patients, 84% were predicted as diseased (BC) patients and 16% predicted as healthy, probably indicating the response to therapy. The sensitivity of the model was high (89.5%) as the neoadjuvant and BC patients were included as true positives and specificity was 87% with an overall accuracy of 79.8%.

Serum metabolic profiles of BC patients on NAC were studied using LC-MS and NMR to identify biomarkers that can predict response to this therapy. A prediction model was built with correct identification in 80% of patients showing incomplete response to NAC^6^. Another study examined the metabolic changes in BC tissues with use of NAC also using MRS. All patients exhibited a significant metabolic response to neoadjuvant chemotherapy differentiating between the pre-treatment and post-treatment spectra with an accuracy of 87.9% by PLS-DA^34^.

### Pathway Analysis

Our study found altered levels of fatty acids (FA) and esters of FAs, alkanes and methylated alkanes/hydrocarbons, aromatic hydrocarbons, amines and amides, carbonyl compounds and sugars in serum of breast cancer patients compared with healthy controls. Moreover pathway analysis on Metaboanalyst revealed disturbances in the following metabolic pathways: galactose metabolism, starch and sucrose metabolism, amino sugar and nucleotide sugar metabolism, fructose and mannose metabolism, fatty acid biosynthesis and purine metabolism. Of these pathways galactose metabolism and starch and sucrose metabolism showed significant alteration (Table S10 of supplementary information).

Altered levels of D-galactose, D-fructose and mannose have been reported in our study. Galactose enters glycolysis through the Leloir pathway, which occurs at a significantly lower rate than glucose entry into glycolysis^35^. It then enters the pentose phosphate pathway to produce nucleotides and phospholipids and thus aid in tumor cell proliferation^35, 36^. Liu *et al*. experimenting on pancreatic cancer cells also reported that fructose caused tumor cell growth in a similar fashion^37^. Up regulation of fructose/mannose metabolism in breast cancer cells has also been reported with increased levels of D-fructose and D-mannose, indicative of an aggressive tumor with an unfavorable prognosis^38^.

Fatty acids and their esters (e.g. octadecanoic acid, heptadecanoic acid, tetradecanoic acid, eicosanoic acid, cis vaccenic acid) showed altered levels in breast cancer patients in our study. Several studies^39–41^ have highlighted the importance of fatty acid oxidation and hence fatty acid metabolism in metabolic transformation of tumor cells^42, 43^ conferring a growth or survival advantage^44^.

Similarly, studies have also reported high concentrations of volatile organic metabolites (VOMs) differentiating between healthy controls and breast cancer patients^14, 33, 45, 46^. Alkanes, methylated alkanes, alcohols, aldehydes, ketones, pentanes or olefins have also been identified as breast cancer markers. Increased oxidative stress results in production of free radicals (ROS) which cause damage to lipids, proteins, DNA and other macromolecules. This in turn generate volatile metabolites in cancer tissues^14, 47^. In addition to oxidative stress, increased levels of cytochrome P (CYP) 450 enzymes resulting in altered estrogen metabolism has also been reported as an additional mechanism for production of organic metabolites in breast diseases^32^. P450 enzymes can also promote biotransformation of alkanes, alkenes, and aromatic compounds^48^.

As previously mentioned most of the metabolomics studies employed NMR-based techniques for analysis^6, 7, 9, 19, 28, 34^, while some of GC-MS based techniques used targeted approaches for identification of metabolites ^11, 12, 15, 31^. Therefore comparison of our metabolites with other studies was a major limitation. However, regarding metabolite identification, we found up-regulation of dimethyldodecane, galactose and α-glyceryl stearate in BC samples as compared to healthy controls. Elevated levels of glucopyranoside, tetradecane, mannose and benzene 1,2 dicarboxylic acid were found to differentiate between different grades, stages and neoadjuvant status.

## Conclusion

This study showed that GC-EI-MS is a simple, sensitive and less invasive method that can be used for the establishment of serum metabolomic profile distinguishing between breast cancer patients and healthy controls with a sensitivity of 96% and specificity of 100% on external validation of PLS-DA model. Moreover, our serum metabolomic approach reports differences in metabolic profile within different grades and stages of breast cancer, a knowledge that can be exploited in diagnosis as well as during treatment. This can revolutionize cancer detection as well as treatment if early grades and stages could be monitored effectively. Studies with larger sample size will be required to validate the results and determine the metabolic differences among different grades and stages.

## Electronic supplementary material

Serum Metabolomic Profiles for Breast Cancer Diagnosis, Grading and Staging by Gas Chromatography-Mass Spectrometry

Dataset 1
