# Supplementary material for: Serum Metabolomic Profiles for Breast Cancer Diagnosis, Grading and Staging by Gas Chromatography-Mass Spectrometry
Source: Sci Rep. 2017 May 11;7:1715. doi: 10.1038/s41598-017-01924-9 (PMC5431835; doi:10.1038/s41598-017-01924-9)
Supplement: Supplementary file 1 — Serum Metabolomic Profiles for Breast Cancer Diagnosis, Grading and Staging by Gas Chromatography-Mass Spectrometry [file 41598_2017_1924_MOESM1_ESM.pdf]

Serum Metabolomic Profiles for Breast Cancer Diagnosis, Grading and Staging by Gas Chromatography-Mass Spectrometry

Naila Irum Hadi[1] , Qamar Jamal[1], Ayesha Iqbal[2], Fouzia Shaikh[1], Saleem Somroo[3], Syed Ghulam Musharraf [2 4]*

* Corresponding author: Syed Ghulam Musharraf musharraf1977@yahoo.com

[1] Department of Pathology, Ziauddin University, Clifton Karachi – 75600, Pakistan.

[2] Dr. Panjwani Center for Molecular Medicine and Drug Research, International Center for Chemical and Biological Sciences, University of Karachi, Karachi – 75270, Pakistan

[3] Breast Clinic, Surgical Ward 2, Jinnah Postgraduate Medical Center (JPMC), Karachi – 75510, Pakistan

[4] H.E.J. Research Institute of Chemistry, International Center for Chemical and Biological Sciences, University of Karachi, Karachi-75270, Pakistan.

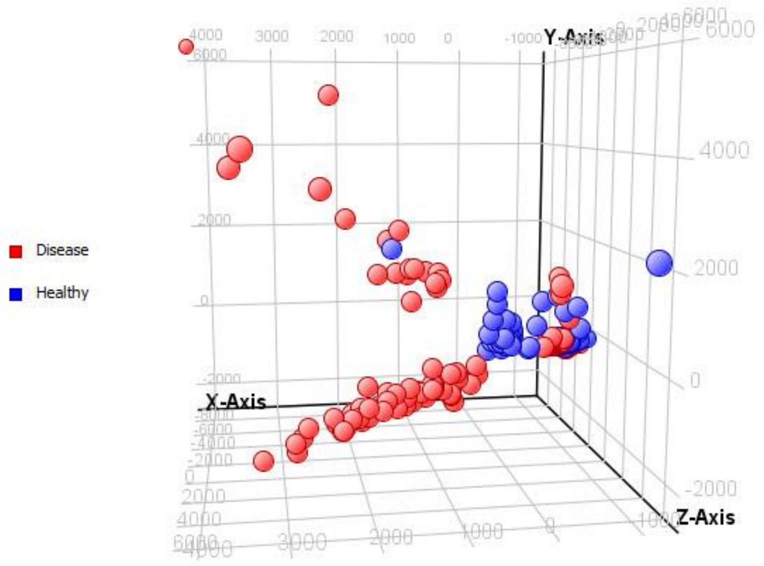

Figure S1. Three-dimensional Principal Component Analysis score plot of all 424 metabolites expressed in healthy and BC serum samples showing variation between the two groups.

Table S1. List of metabolites (07 entities) distinguishing between healthy controls (H) and BC patients (Ds) at fold change > 1.5 and p < 0.05

**Compounds**	**Probable Source**	**Retention time (RT) (min)**	**P-value**	**FC**	**Log FC**
Tetradecane (CAS ID 629-59-4)	Not available	10.97	2.87×10^{-12}	-1.74	-0.80
Glucopyranoside (CAS ID 19159-25-2)	Endogenous	23.4	1.24×10^{-21}	-2.07	-1.05
Methyl stearate (CAS ID 112-61-8)	Endogenous	18.71	6.3×10^{-08}	-1.56	-0.64
Dodecane (CAS ID 61141-72-8)	Endogenous	12.88	1.18×10^{-08}	1.52	0.61
1-4-substituted Benzene* (CAS ID 999478-18-9)	Not available	6.29	5.75×10^{-07}	-1.508	-0.59
Galactose (CAS ID 6736-94-3)	Endogenous	17.58	3.55×10^{-11}	1.69	0.76
α-Glyceryl stearate (CAS ID 1188-75-6)	Endogenous	24.04	2.30×10^{-15}	1.86	0.89

*1(1-Methoxycarbonyethyl)-4-(2-methyl-2-trimethylsilyl-oxypropyl) Benzene
CAS No. – Chemical Abstracts Service Number, FC – Fold Change, H – healthy controls, Ds – diseased, BC – breast cancer

Fold change (FC) with a positive value indicates that the concentration of certain metabolite is up-regulated in breast cancer compared with healthy controls. FC with a negative value indicates that the concentration of certain metabolite is down-regulated in breast cancer compared with healthy controls.

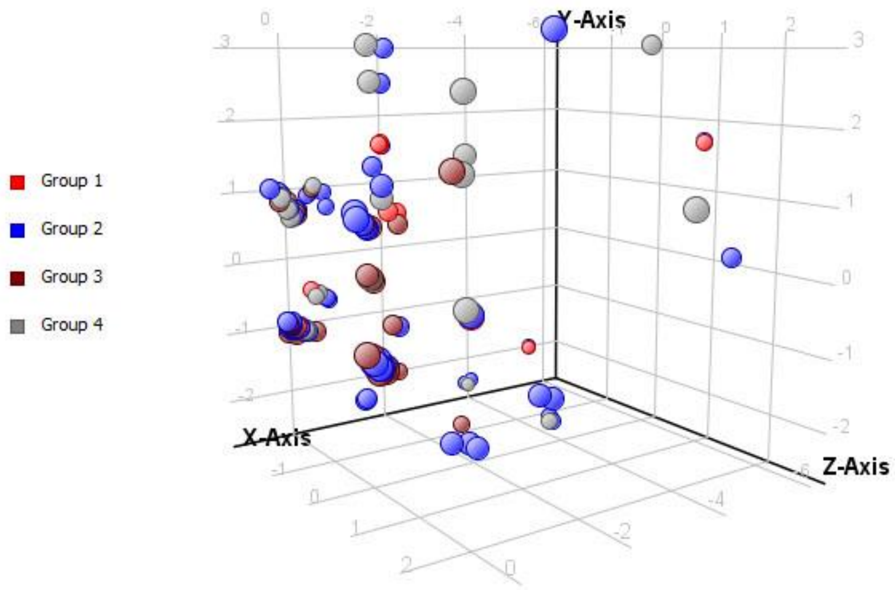

Figure S2. Three-dimensional Principal Component Analysis score plot of statistically significant metabolites in 155 healthy serum samples showing no variation between different age groups.

Group 1: 20-35 years, Group 2: 36-50 years, Group 3: 51-65 years, Group 4: 66-80 years

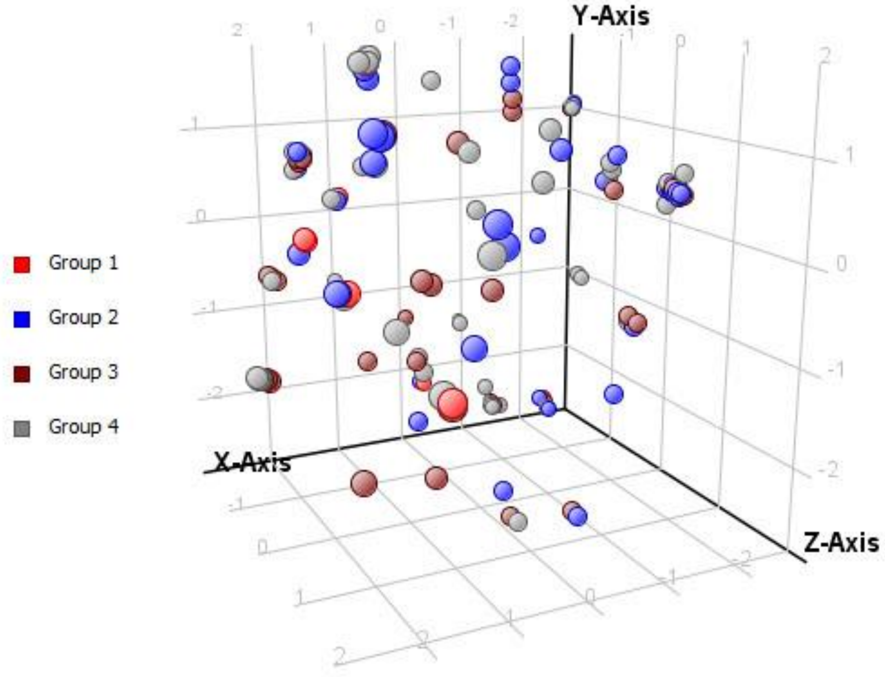

Figure S3. Three-dimensional Principal Component Analysis score plot of statistically significant metabolites in 152 BC serum samples showing no variation between different age groups.

Group 1: 20-35 years, Group 2: 36-50 years, Group 3: 51-65 years, Group 4: 66-80 years

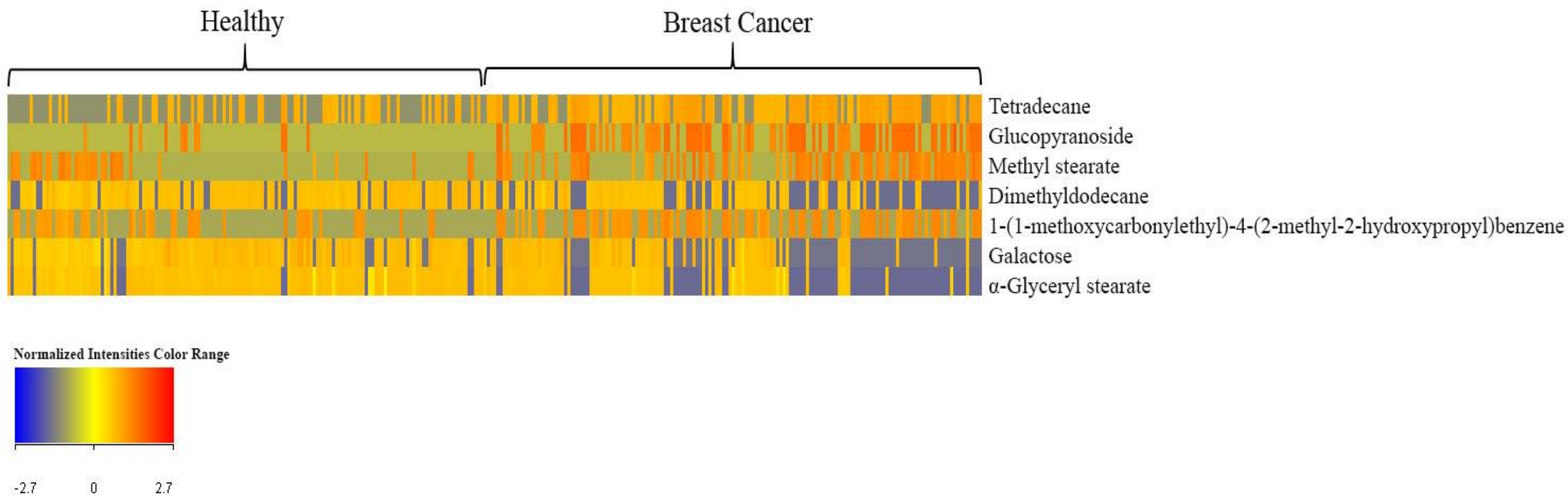

Figure S4. Heat map visualization of all samples based on 7 differential entitie

Table S2. List of metabolites (18 entities) distinguishing between healthy controls (H) and three grades of breast cancer (G I, II & III) at fold change > 2 and $p < 0.05$

Compounds	Probable Source	RT (min)	P-value	FC (H VS GI)	Log FC (H VS GI)	FC (H VS GII)	Log FC (H VS GII)	FC (H VS GIII)	Log FC (H VS GIII)
Dibenzo[c,e]thiin-2-thione (CAS ID 999257-86-3)	Not available	8.01	2.2 E-13	2.38	1.25	1.68	0.75	1.37	0.45
Tetradecane (CAS ID 629-59-4)	Not available	10.97	1.1 E-16	2.09	1.06	1.99	0.99	1.45	0.54
Heptadecanoic acid (CAS ID 55517-58-3)	Exogenous	18.9	4,4 E-13	2.79	1.48	1.42	0.52	1.32	0.4
Menthone (CAS ID 10458-14-7)	Not available	7.55	2.1 E-11	2.5	1.32	1.51	0.59	1.17	0.22
Fructose (CAS ID 19126-98-8)	Endogenous	15.99	3.9 E-20	2.84	1.51	1.91	0.93	1.46	0.55
Eicosanoic acid (CAS ID 55530-70-6)	Exogenous	21.4	2.6 E-15	2.56	1.36	1.64	0.71	1.52	0.61
Glucopyranoside (CAS ID 19159-25-2)	Endogenous	23.4	2.8 E-29	2.72	1.44	2.25	1.17	1.91	0.93
Mannose (CAS ID 6736-99-8)	Endogenous	17.6	1.3 E-18	2.3	1.2	1.97	0.97	1.61	0.68
5-methyl-2-(1-methyl-ethyl) Cyclohexanone (CAS ID=89-80-5)	Endogenous	7.58	1.02 E-08	2.11	1.08	1.49	0.58	1.16	0.21
Benzene-1, 2-dicarboxylic acid (CAS ID 117-81-7)	Exogenous	22.3	2.4 E-06	2.33	1.22	2.1	1.01	1.42	0.5
Citronellol (CAS ID 999258-24-3)	Endogenous	9.8	1.8 E-10	2.57	1.36	1.37	0.46	1.17	0.23
(2-chloroethyl) sulfonyl benzene (CAS ID 938-09-0)	Not available	14.83	2.2 E-17	2.23	1.16	1.99	0.99	1.41	0.49
Hexadecane (CAS ID 544-76-3)	Exogenous	13.46	5.2 E-11	2.76	1.46	1.28	0.35	1.19	0.25
(3,7-dimethyl-6-octenyl)hydroxyl	Endogenous	9.82	4.96 E-08	-2.29	-1.19	-1.32	-0.39	-1.11	-0.15

(CAS ID 18419-09-5)									
α-Glyceryl stearate (CAS ID 1188-75-6).	Endogenous	24.04	4.26 E-23	-2.68	-1.42	-2.13	-1.09	-1.54	-0.62
71.0@10.0		10	1.99 E-09	2.41	1.27	1.32	0.4	1.27	0.35
250.0@6.5:2		6.5	4.32 E-19	3.79	1.92	1.43	0.51	1.27	0.35
71.0@12.299997		12.3	1.47 E-17	3.67	1.88	1.23	0.29	1.33	0.41

CAS ID – Chemical Abstracts Service , RT (min) – Retention time in minutes, FC – Fold Change, G1 – grade I, GI – grade II, G1II – grade III, G1V – grade IV

Cpd: 71.0@10.0

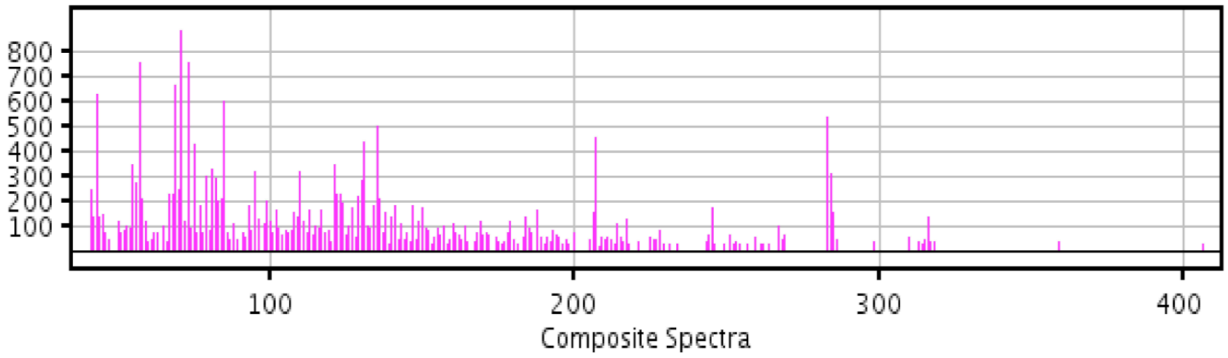

Cpd: 71.0@12.299997

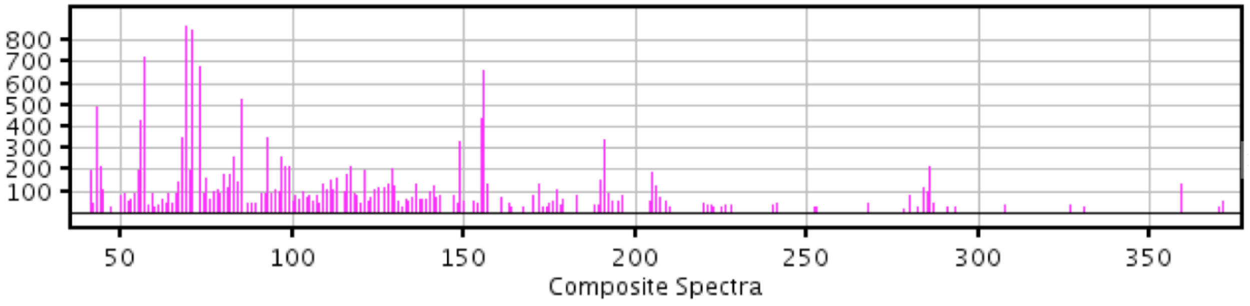

Cpd: 250.0@6.52

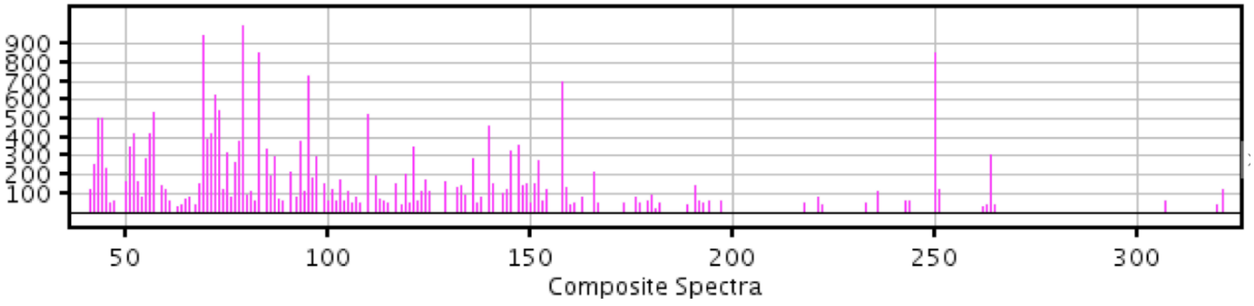

Figure S5. The EI/MS spectra of unidentified compounds that are statistically differentially expressed between healthy controls and grades (I, II, III) of breast cancer (BC) patients.

Table S3. Summary of Tukey HSD post hoc test for healthy controls versus grades of breast cancer. Entities or metabolite found to be differentially expressed are represented in gray boxes and significantly expressed, while entities found not to be differentially expressed are represented in orange boxes.

**Group Name**	**Grade II**	**Grade I**	**Grade III**	**Healthy**
**Grade II**	18	8	3	10
**Grade I**	10	18	15	18
**Grade III**	15	3	18	13
**Healthy**	8	0	5	18

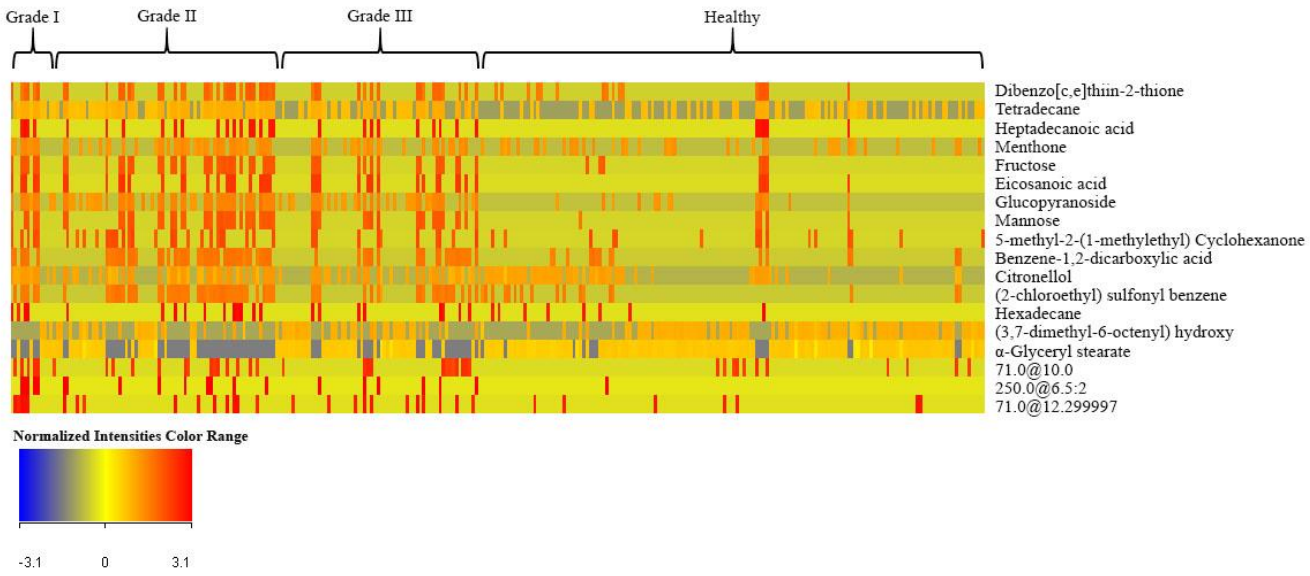

Figure S6. Heat map visualization based on 18 significantly expressed metabolites differentiating between healthy controls and different grades (I-III) of BC.

Table S4a: List of metabolites (20 entities) distinguishing between healthy controls (H) and four stages of breast cancer

Compounds	Probable Source	CAS No.	RT(min)	P-value
Dibenzo[c,e]thiin-2-thione	Not available	999257-86-3	8.02	8.16 E-12
Tetradecane	Not available	629-59-4	10.98	1.52 E-11
Fructose	Endogenous	19126-98-8	15.99	5.88 E-15
1,8-Diphenyl-3,4,10,11-tetra-hydro[1,4]dioxino diisoquinoline	Not available	57315-73-8	29.9	6.62 E-22
Glucopyranoside	Endogenous	19159-25-2	23.4	8.31 E-23
Methyl stearate	Endogenous	112-61-8	18.71	1.57 E-09
Mannose	Endogenous	6736-99-8	17.63	6.71 E-19
Benzene-1, 2-dicarboxylic acid	Exogenous	117-81-7	22.3	2.92 E-13
(2-chloroethyl) sulfonyl benzene	Not available	938-09-0	14.83	4.25 E-14
cis-Vaccenic acid	Endogenous	506-17-2	18.84	0.001876
Phytane	Not available	638-36-8	16.26	6.84 E-06
Dipyrrolo[1,2-a:2',1'-c]pyrazine	Not available	211-55-2	11.48	8.49 E-17
Sulfurous acid, 2-ethylhexyl undecyl ester	Exogenous	999571-16-6	12.79	4.98 E-05
Dimethyldodecane	Not available	6114172-8	12.87	2.28 E-13
DNOP*	Exogenous	117-84-0	22.16	1.93 E-09
hept-1-en-6-yne-3,4-diol	Not available	999174-66-1	9.43	1.60 E-04
Galactose	Endogenous	6736-94-3	17.59	5.64 E-14
α-Glyceryl stearate	Endogenous	1188-75-6	24.04	5.14 E-19
Pentanethioamide	Not available	101402-31-7	7.71	5.9 E-15
73.0@7.3000016			7.3	1.3 E-06

DNOP* - (1, 2 Benzenedicarboxylic acid, bis (2-ethylhexyl) ester)
CAS No. – Chemical Abstracts Service Number, RT (min) – Retention time in minutes, H – healthy controls

Table S4b: List of metabolites (20 entities) distinguishing between healthy controls (H) and four stages of breast cancer (S I, II, III & IV) at fold change > 2 and p < 0.05.

**Compounds**	**FC** (H VS SI)	**Log FC** (H VS SI)	**FC** (H VSSII)	**Log FC** (H VS SII)	**FC** (H VS SIII)	**Log FC** (H VS SIII)	**FC** (H VS SIV)	**Log FC** (H VS SIV)
Dibenzo[c,e]thiin2-thione	-1.18	-0.24	2.53	1.34	1.37	0.45	1.09	0.12
Tetradecane	1.16	0.21	2.04	1.03	1.72	0.78	1.45	0.53
Fructose	-1.07	-0.1	2.64	1.4	1.56	0.64	1.38	0.47
1,8-Diphenyl-3,4,10,11-tetrahydro[1,4]dioxino diisoquinoline	1.55	0.64	2.94	1.55	1.73	0.79	1.27	0.35
Glucopyranoside	1.23	0.29	2.55	1.35	2.02	1.01	1.99	0.99
Methyl stearate	2.03	1.02	1.88	0.91	1.42	0.5	1.06	0.08
Mannose	-1.06	-0.08	2.97	1.57	1.58	0.66	1.40	0.49
Benzene-1, 2dicarboxylic acid	1.27	0.35	2.38	1.25	1.61	0.68	1.21	0.27
(2-chloroethyl) sulfonyl benzene	1.26	0.34	2.49	1.32	1.56	0.64	1.29	0.36
cis-Vaccenic acid	2.0	1.0	1.09	0.13	1.14	0.19	1.17	0.23
Phytane	-2.2	-1.14	1.04	0.06	-1.13	-0.18	-1.49	-0.58
Dipyrrolo[1,2a:2',1'-c]pyrazine	-1.03	-0.04	2.86	1.51	1.41	0.49	1.49	0.58
Sulfurous acid, 2-ethylhexyl undecyl ester	2.55	1.35	1.04	0.05	-1.25	-0.33	-1.30	-0.38
Dimethyldodecane	-2.41	-1.27	-2.08	-1.05	-1.32	-0.40	-1.18	-0.24
DNOP*	-2.11	-1.08	-1.47	-0.55	-1.43	-0.52	-1.57	-0.65
hept-1-en-6-yne3,4-diol	2.41	1.26	1.05	0.07	1.09	0.13	1.12	0.16
Galactose	-1.17	-0.23	-2.54	-1.34	-1.49	-0.58	-1.34	-0.42
α-Glyceryl stearate	-1.25	-0.32	-2.86	-1.51	-1.64	-0.72	-1.35	-0.43
Pentanethioamide	2.84	1.51	1.03	0.05	1.61	0.68	1.89	0.92
73.0@7.3000016	3.19	1.67	1.007	0.011	1.24	0.311	-1.15	-0.19

DNOP* - (1, 2 Benzenedicarboxylic acid, bis (2-ethylhexyl) ester), FC – fold change, H – healthy controls, S I – stage I of BC, S II - stage II of BC, S III - stage III of BC, S IV - stage IV of BC

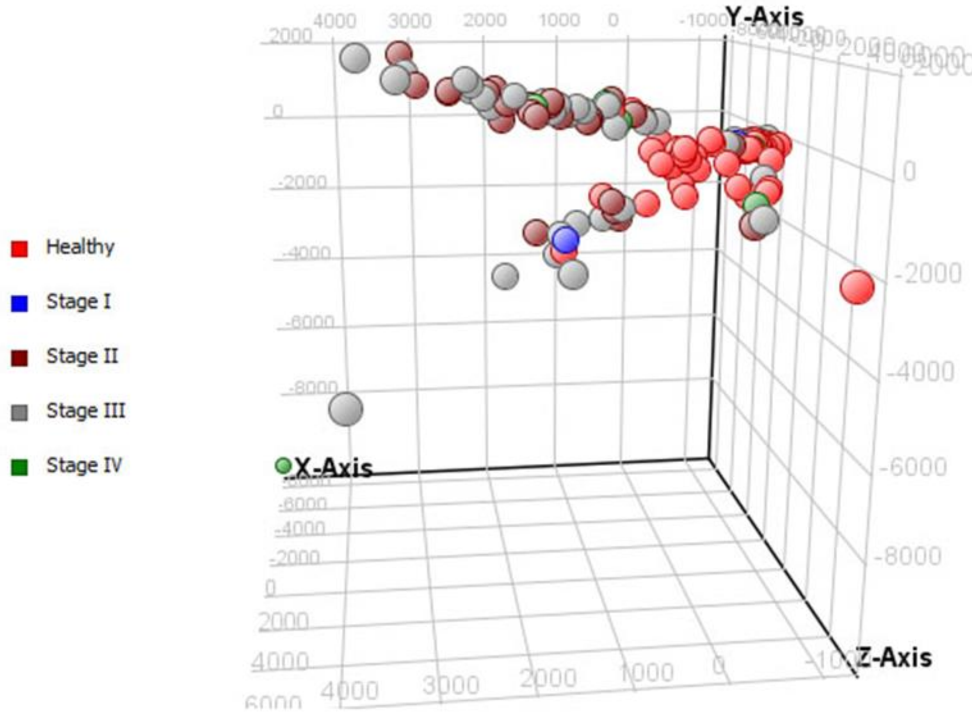

Figure S7: Three-dimensional Principal Component Analysis score plot of all 424 metabolites in healthy and BC serum samples showing clustering of the two groups with little variation between different stages.

Cpd: 73.0@7.3000016

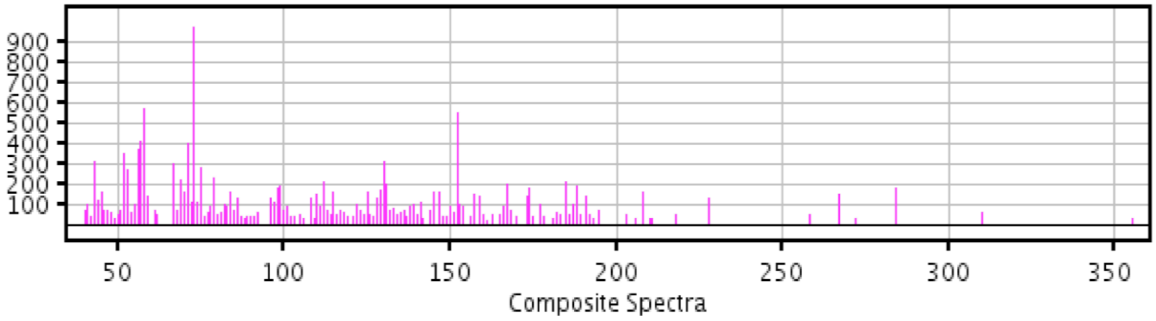

Figure S8.The EI/MS spectrum of unidentified compound that is statistically differentially expressed between healthy controls and stages (I, II, III, IV) of breast cancer (BC).

Table S5. Summary of Tukey HSD post hoc test for healthy controls versus stages of breast cancer. Entities or metabolite found to be differentially expressed are represented in gray boxes and significantly expressed, while entities not differentially expressed are represented in orange boxes.

**Group Name**	**Stage IV**	**Stage I**	**Healthy**	**Stage II**	**Stage III**
**Stage IV**	20	7	10	9	5
**Stage I**	13	20	10	18	15
**Healthy**	10	10	20	13	10
**Stage II**	11	2	7	20	8
**Stage III**	15	5	10	12	20

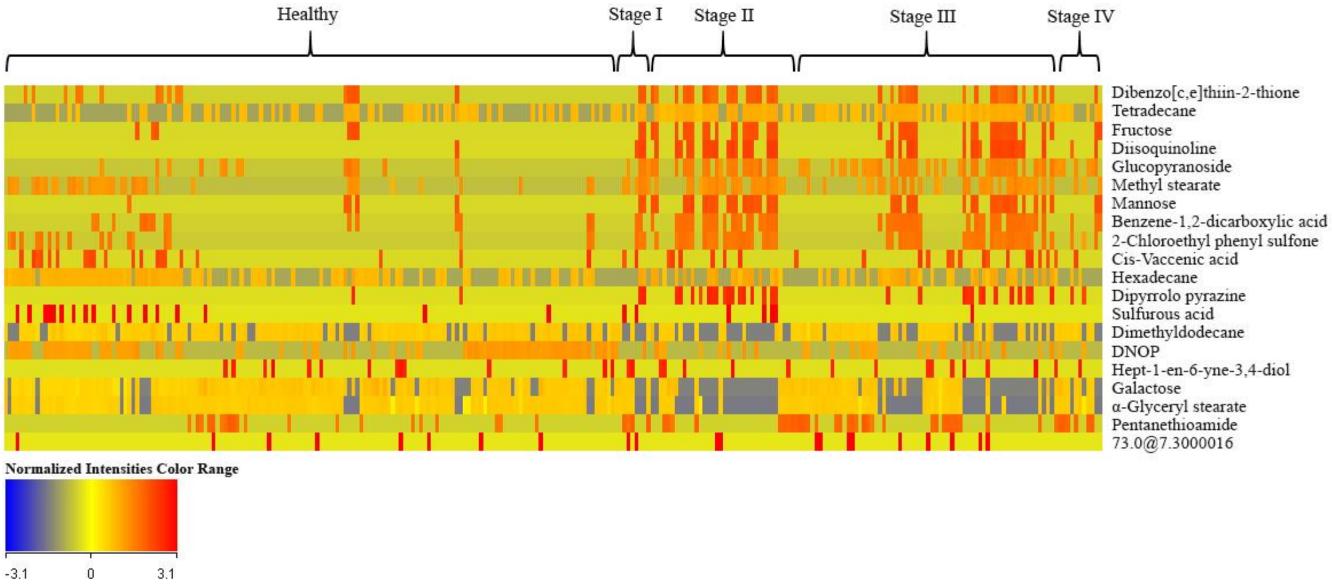

Figure S9. Heat map visualization of all analyzed samples based on normalized intensities of 20 significantly expressed metabolites differentiating between healthy controls and different stages (I-IV) of BC.

Table S6. List of metabolites (14 entities) distinguishing between healthy controls (H) and three sub-categories of stage III (A, B & C) of breast cancer at fold change > 2 and p < 0.05.

Compounds	Probable Source	RT (min)	P-value	FC (H VS IIIA)	Log FC (H VS IIIA)	FC (H VS IIIB)	Log FC (H VS III B)	FC (H VS IIIC)	Log FC (H VS IIIC)
Dibenzo[c,e]thiin-2-thione (CAS ID 999257-86-3)	Not Available	8.1	1.4 E-10	1.33	0.41	2.69	1.43	1.21	0.28
Fructose (CAS ID 19126-98-8)	Endogenous	16	2.6 E-15	1.68	0.75	2.73	1.45	1.42	0.51
Glucopyranoside (CAS ID 19159-25-2)	Endogenous	23.41	2.4 E-25	2.12	1.09	2.42	1.27	2.24	1.16
Mannose (CAS ID 6736-99-8)	Endogenous	17.55	8.1 E-17	1.74	0.79	2.83	1.50	1.46	0.55
Benzene-1, 2-dicarboxylic acid (CAS ID 117-81-7)	Exogenous	22.29	1.5 E-14	1.97	0.97	2.46	1.29	1.11	0.14
Tetradecanoic acid (CAS ID 18603-17-3)	Endogenous	16.1	9.2 E-12	1.34	0.42	3.68	1.88	-1.09	-0.13
1-methylene-3methyl-Butane-1,3-diol (CAS ID 117201-96-4)	Not Available	8.11	5.4 E-14	1.30	0.38	3.32	1.73	1.17	0.23
Dimethyldodecane (CAS ID=61141-72-8)	Not Available	12.83	1.6 E-08	-1.49	-0.58	-2.38	-1.25	1.13	0.18
Galactose (CAS ID 6736-94-3)	Endogenous	17.59	1.5 E-11	-1.69	-0.76	-2.45	-1.29	-1.09	-0.13
α-Glyceryl stearate (CAS ID 1188-75-6)	Endogenous	24.05	4.6 E-15	-2.01	-1.01	-2.39	-1.25	-1.2	-0.27
Pentanethioamide (CAS ID=101402-31-7)	Not Available	7.7	8.4 E-09	1.52	0.60	1.15	0.21	2.09	1.06
131.0		15.29	1.7 E-09	1.60	0.68	2.10	1.07	1.28	0.35
57.0		10.1	1.2 E-07	1.37	0.46	2.33	1.22	1.02	0.024
71.0		10	2.2 E-09	1.48	0.57	2.51	1.33	-1.04	-0.06

CAS – Chemical Abstracts Service Number, RT (min) – Retention time in minutes, FC – Fold Change,
H – Healthy controls, S III A – stage III A of BC, S III B - stage III B of BC, S III C - stage III C of BC

Cpd: 71.0@10.0

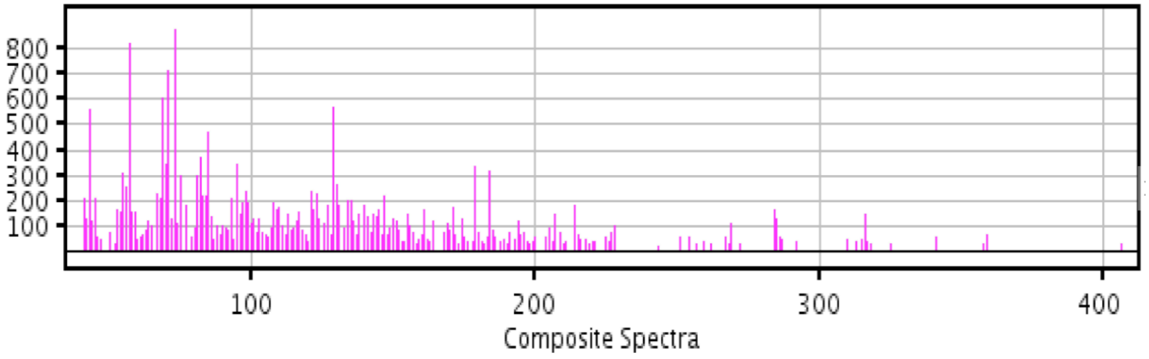

Cpd: 131.0@15.2999935

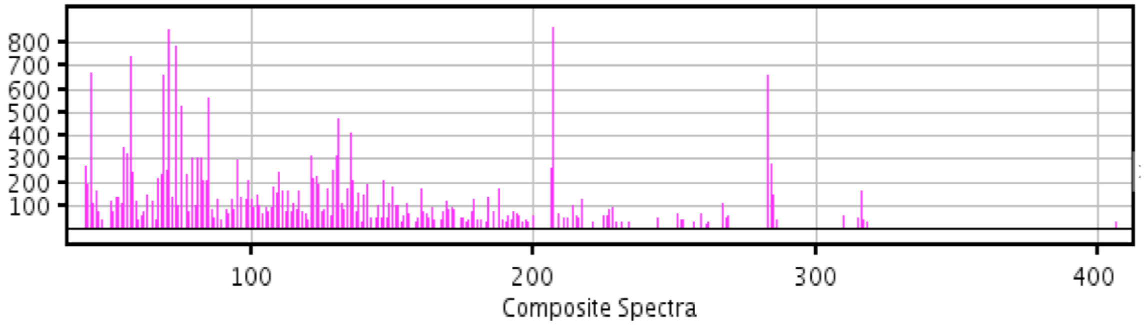

Cpd: 57@10.190003

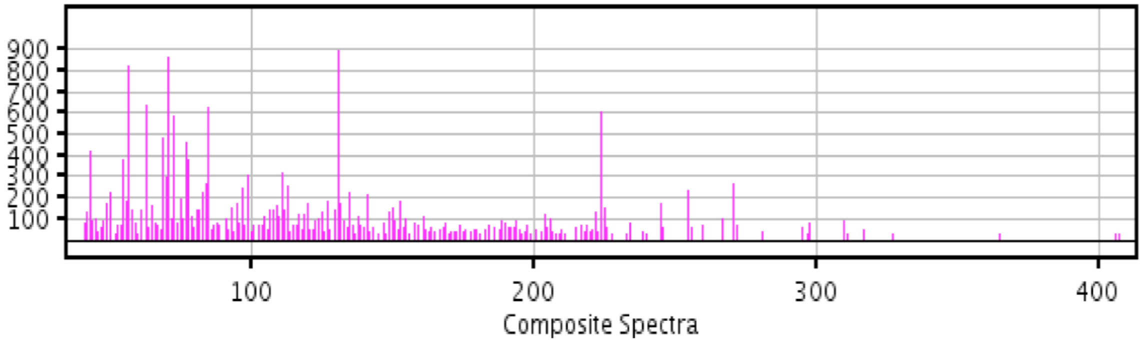

Figure S10.The EI/MS spectra of unidentified compounds that are statistically differentially expressed between healthy controls and subcategories of stage III (A, B, C) of breast cancer (BC) patients.

Table S7. Summary of Tukey HSD post hoc test for healthy controls versus stage III sub-categories of breast cancer. Entities or metabolite found to be differentially expressed are represented in blue boxes and significantly expressed, while entities not differentially expressed are represented in orange boxes.

Group Name	Stage III A	Stage III C	Healthy	Stage III B
**Stage III A**	14	3	7	6
**Stage III C**	11	14	12	13
**Healthy**	7	2	14	14
**Stage III B**	8	1	0	14

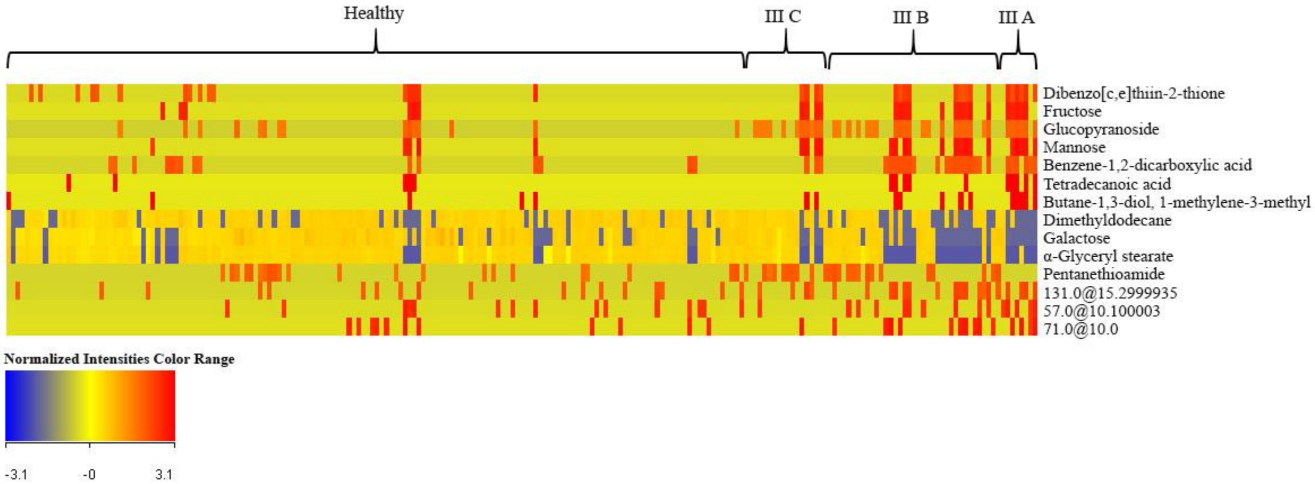

Figure S11. Heat map visualization based on 14 significantly expressed metabolites differentiating between healthy controls and different stage III sub-categories (A-C) of BC.

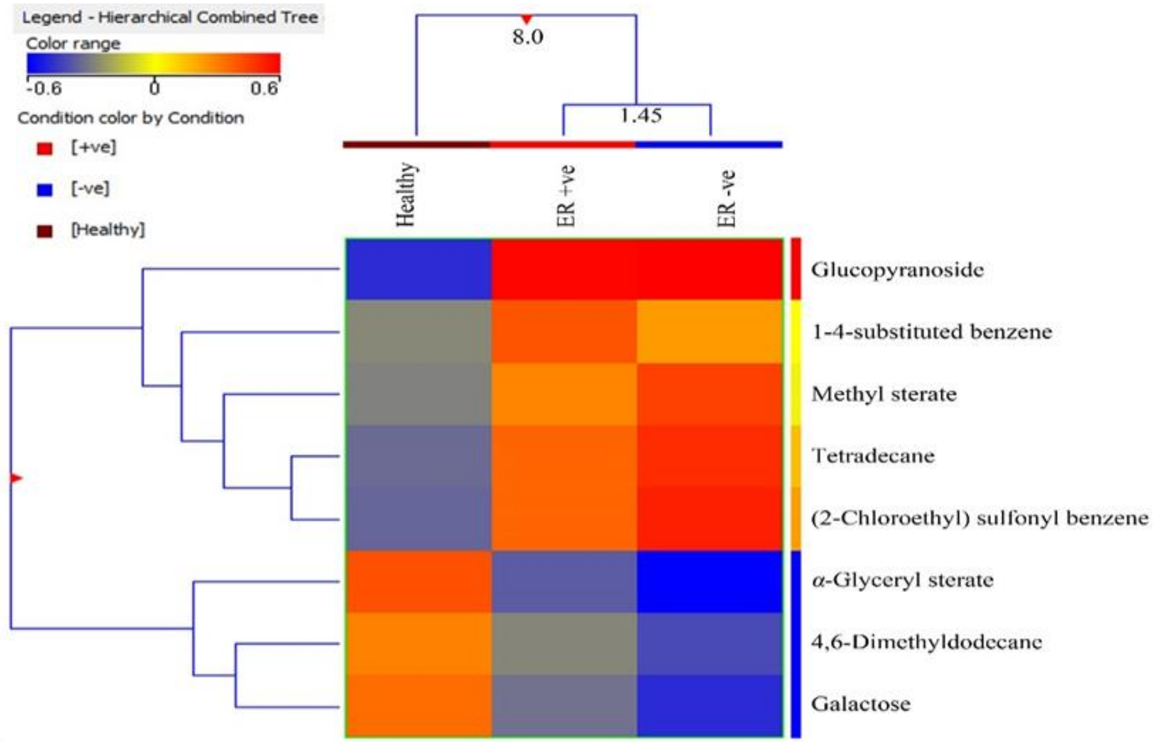

Figure S12a. Dendogram showing comparison of healthy controls and two groups of BC patients i.e. patients with estrogen receptor positive (ER+) BC, and estrogen receptor negative (ER-) BC using normalized intensities of eight significant metabolites ($p<0.05$)

(1-4-substituted benzene, 1(1-Methoxycarbonyethyl)-4-(2-methyl-2-trimethylsilyl oxypropyl) Benzene)

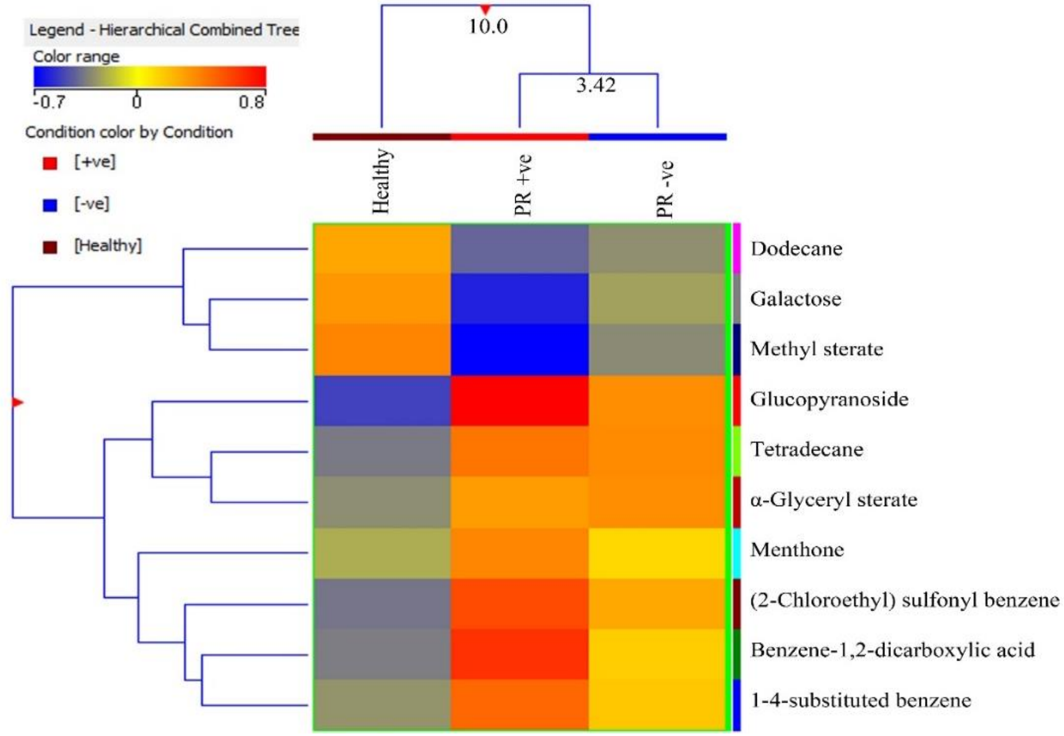

Figure S12b. Dendogram showing comparison of healthy controls and two groups of BC patients i.e. patients with Progesterone receptor positive (PR+) BC, and Progesterone receptor negative (PR-) BC using normalized intensities of ten significant metabolites ($p<0.05$). (*1(1-Methoxycarbonyethyl)-4-(2-methyl-2-trimethylsilyl-oxypropyl) Benzene; PR, Progesterone receptor; +/-, positive/ negative; BC – Breast cancer)

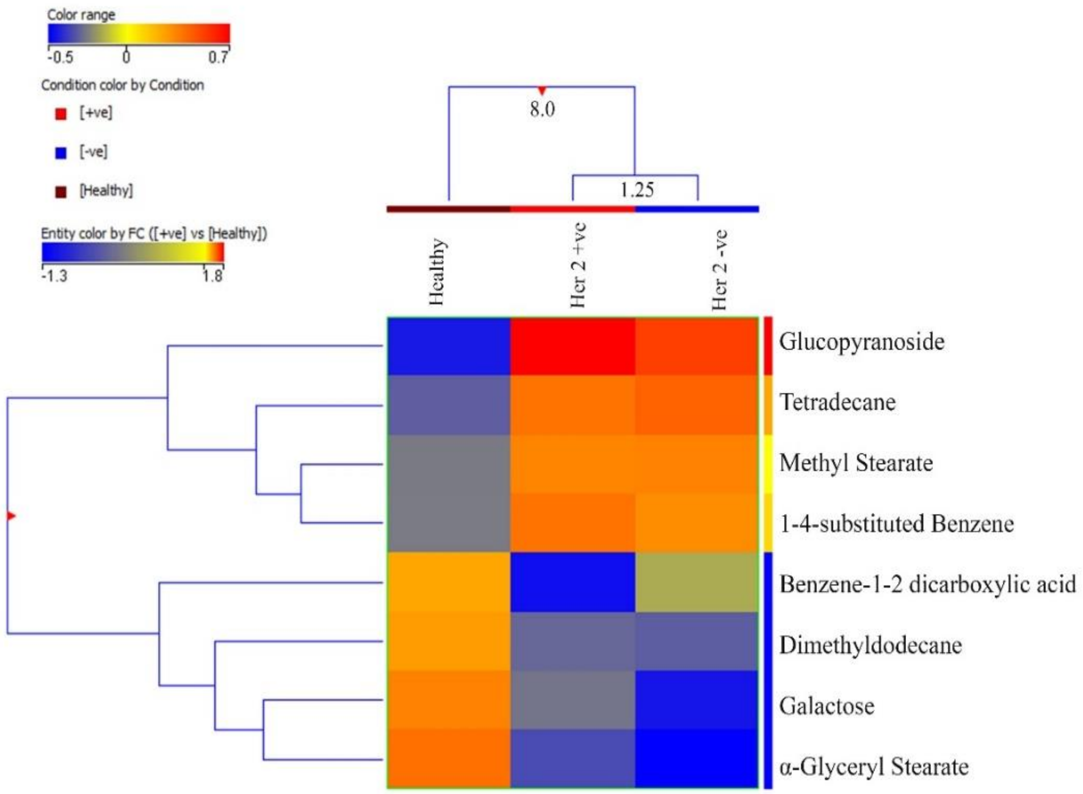

Figure S12c. Dendogram showing comparison of healthy controls and two groups of BC patients i.e. patients with Human epidermal growth factor receptor positive (HER2+) and negative (HER2-) BC using normalized intensities of eight significant metabolites ($p<0.05$). [1-4-substituted Benzene -1(1-Methoxycarbonyethyl)-4-(2-methyl-2-trimethylsilyl-oxypropyl) Benzene]

Table S8. List of metabolites (10 entities) distinguishing between healthy controls (H), breast cancer patients (BC) and BC patients on neoadjuvant therapy (Neo) at fold change 1.5 and $p < 0.05$

**Compounds**	**Probable Source**	**RT** (min)	**P-value**	**FC** (H VS BC)	**Log FC** (H VS BC)	**FC** (H VS Neo)	**Log FC** (H VS Neo)
Tetradecane (CAS ID 629-59-4)	Not Available	10.97	1.8 E-13	1.69	0.76	1.93	0.95
Glucopyranoside (CAS ID 19159-25-2)	Endogenous	23.40	4.5 E-22	2.12	1.08	1.88	0.91
Methyl stearate (CAS ID 112-61-8)	Endogenous	18.71	3.3 E-09	1.5	0.59	1.79	0.84
Benzene-1, 2-dicarboxylic acid (CAS ID 117-81-7)	Exogenous	22.30	3.2 E-13	1.69	0.75	1.92	0.94
(2-Chloroethyl) sulfonyl benzene (CAS ID 938-09-0)	Not Available	14.83	4.8 E-13	1.69	0.76	1.87	0.90
Dimethyldodecane (CAS ID 61141-72-8)	Not Available	12.88	7.1 E-08	-1.49	-0.57	-1.67	-0.74
1-4 substituted benzene* (CAS ID 999478-18-9)	Not Available	6.29	8.9 E-07	1.57	0.65	1.29	0.38
DNOP** (CAS ID 117-84-0)	Exogenous	22.17	9.9 E-07	-1.44	-0.53	-1.61	-0.68
Galactose (CAS ID 6736-94-3)	Endogenous	17.58	4.8 E-13	-1.63	-0.71	-1.98	-0.98
α-Glyceryl stearate (CAS ID 1188-75-6)	Endogenous	24.04	1.1 E-17	-1.79	-0.84	-2.18	-1.12

*1-4 substituted benzene- [1(1-Methoxycarbonyethyl)-4-(2-methyl-2-trimethylsilyl-oxypropyl) Benzene]
**DNOP - (1, 2 Benzenedicarboxylic acid, bis (2-ethylhexyl) ester), FC – fold change, Neo – Breast cancer patients on neoadjuvant therapy, H – Healthy controls, BC – Breast cancer

(FC with a positive value indicates that the concentration of certain metabolite is up-regulated in breast cancer, while FC with a negative value indicates that the concentration of certain metabolite is down-regulated in breast cancer compared with healthy controls.)

Table S9. Summary of Tukey HSD post hoc test for healthy controls versus breast cancer and neoadjuvamt status. Entities or metabolite found to be differentially expressed are represented in blue boxes and significantly expressed, while entities not differentially expressed are represented in orange boxes.

**Group Name**	**Neoadjuvant**	**Healthy**	**Breast cancer**
**Neoadjuvant**	10	9	0
**Healthy**	1	10	10
**Breast cancer**	10	0	10

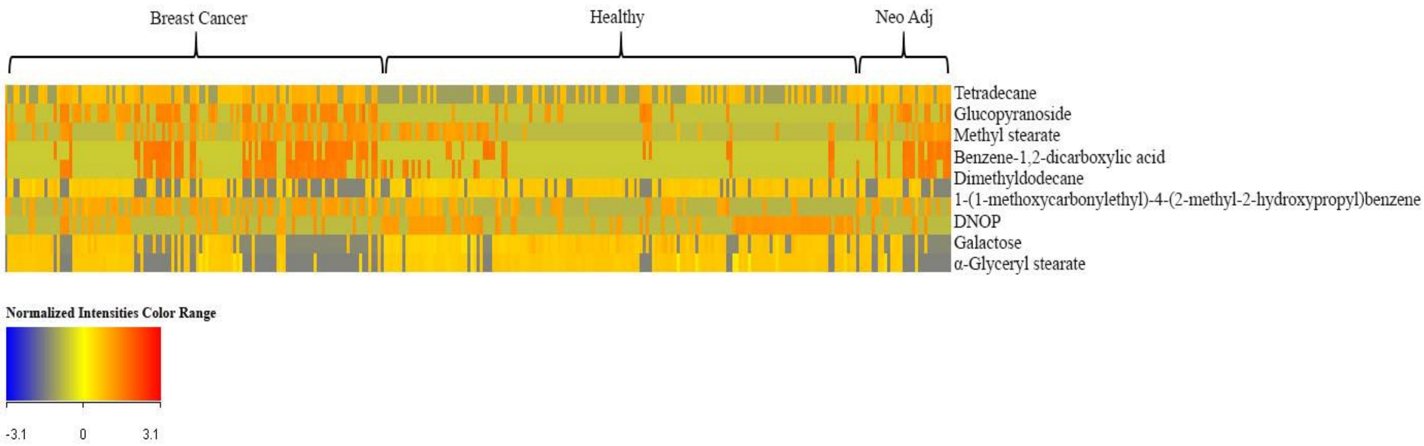

Figure S13. Heat map visualization based on significantly expressed metabolites differentiating between healthy controls, BC patients and patients on neoadjuvant therapy.

Figure S14. Model generated PCA scores scatter plots discriminating among healthy controls and breast cancer based on the seven significance metabolite data.

Sample 1-50 of Healthy

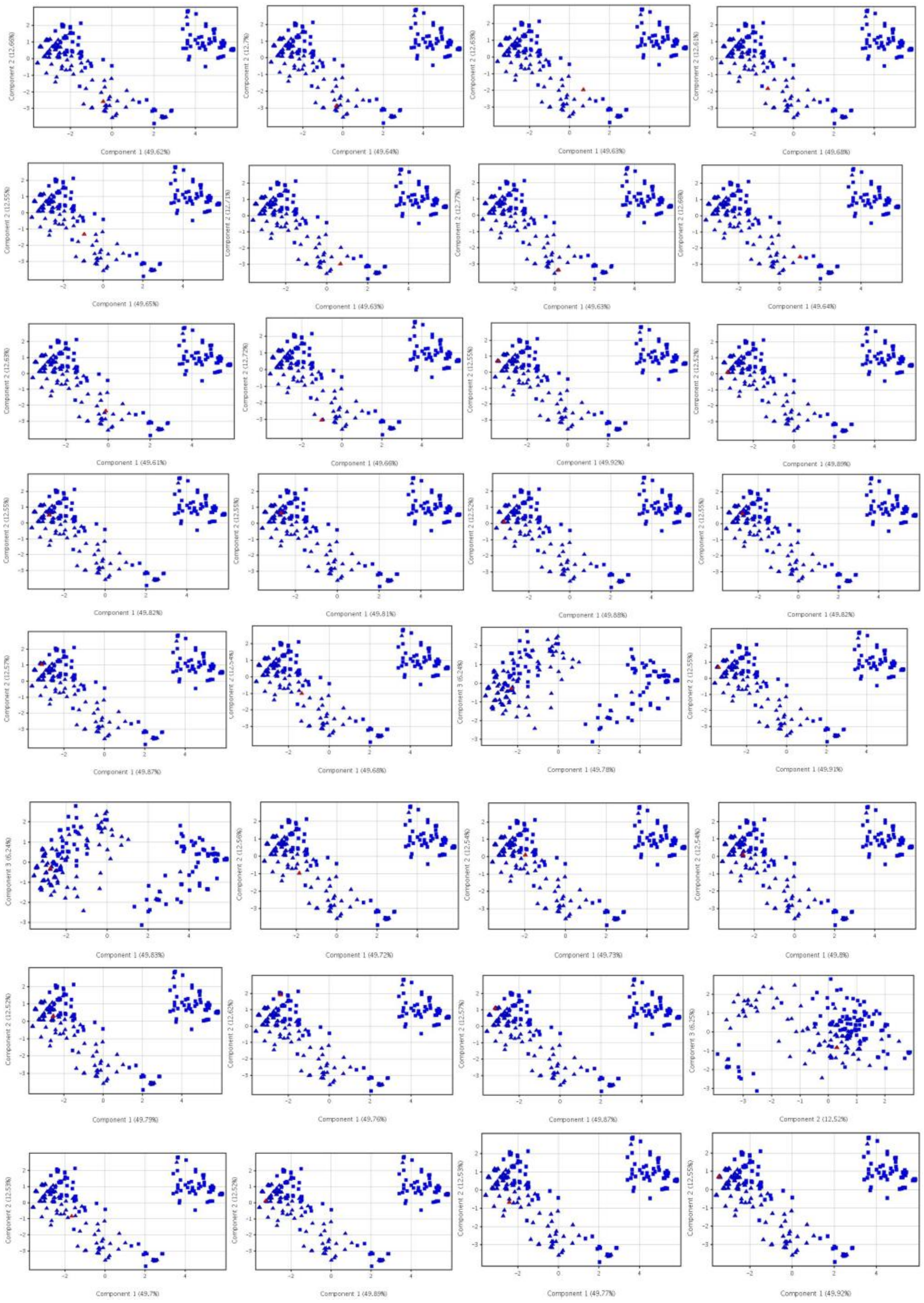

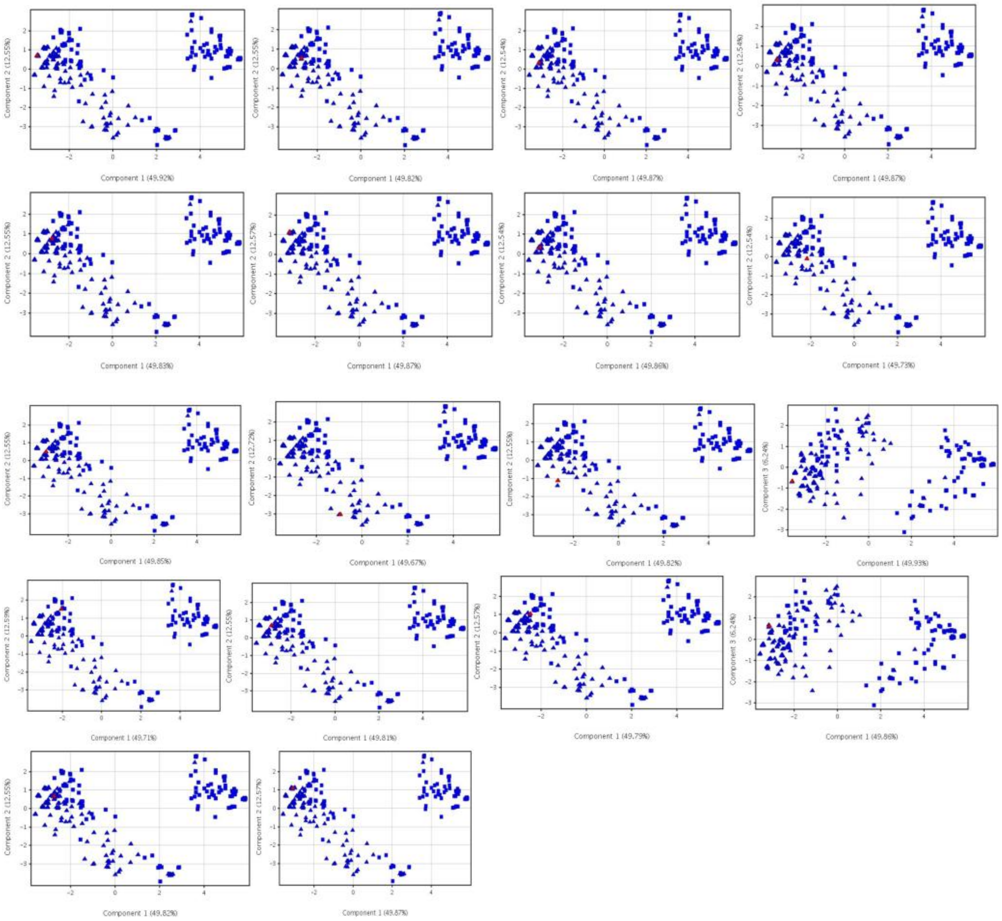

Sample 51-100 of Breast Cancer

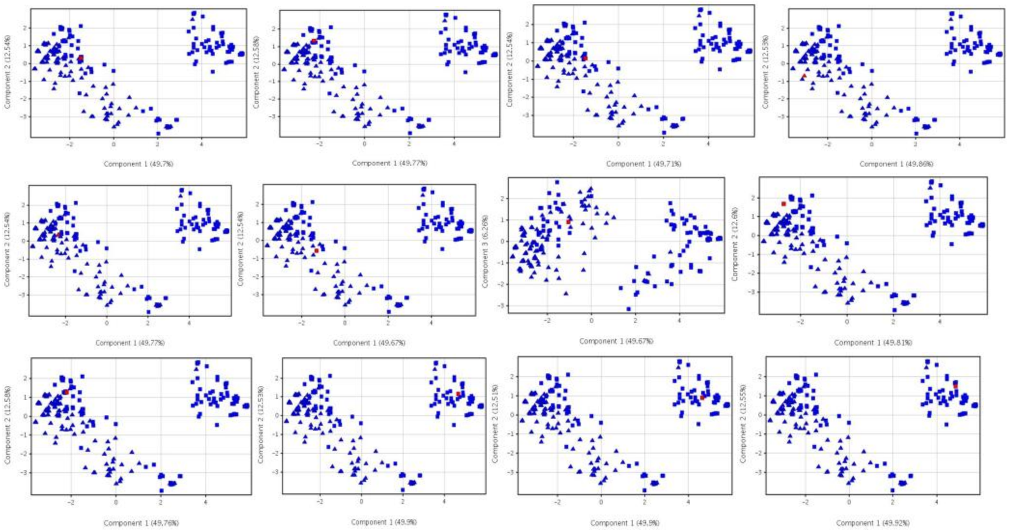

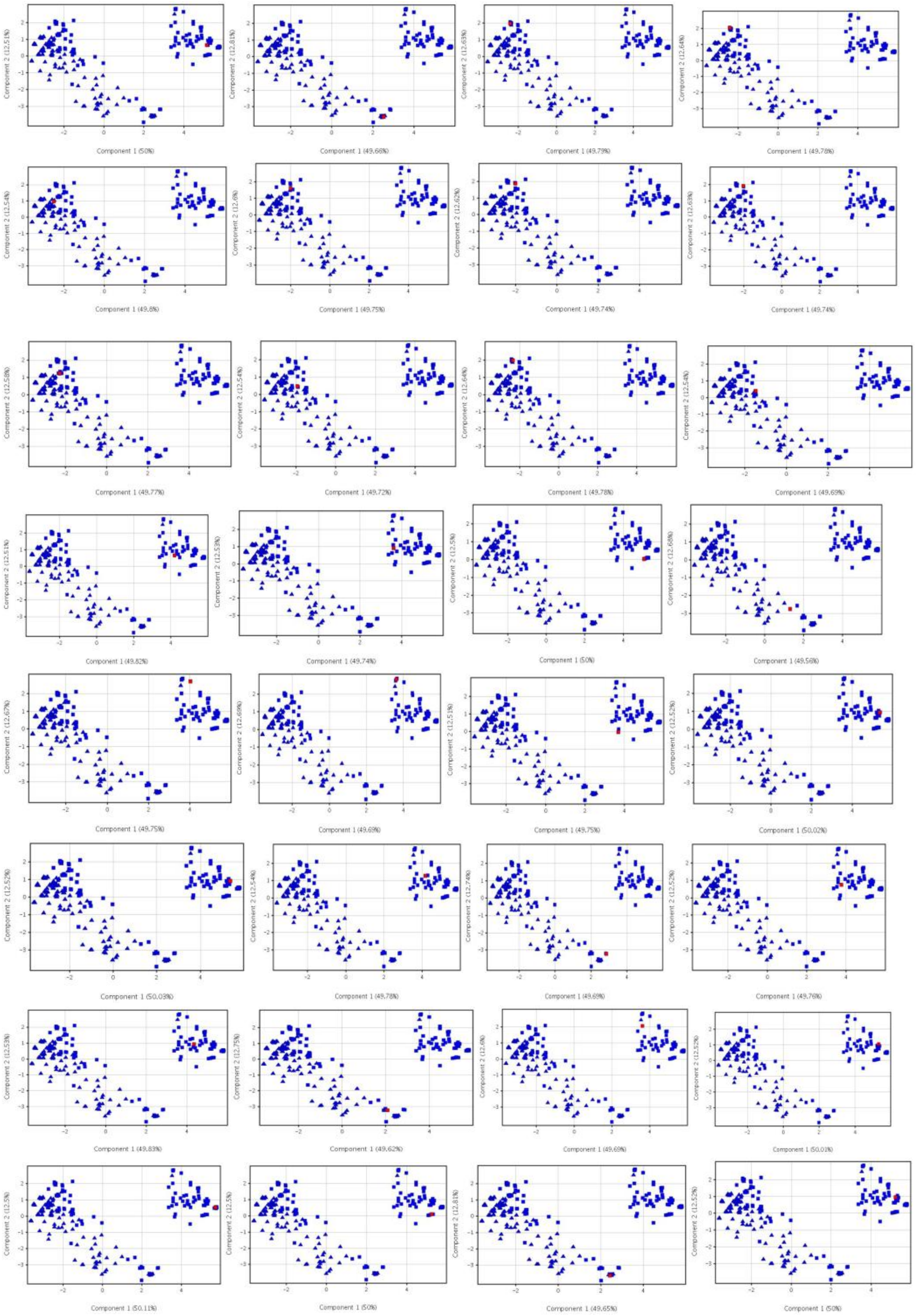

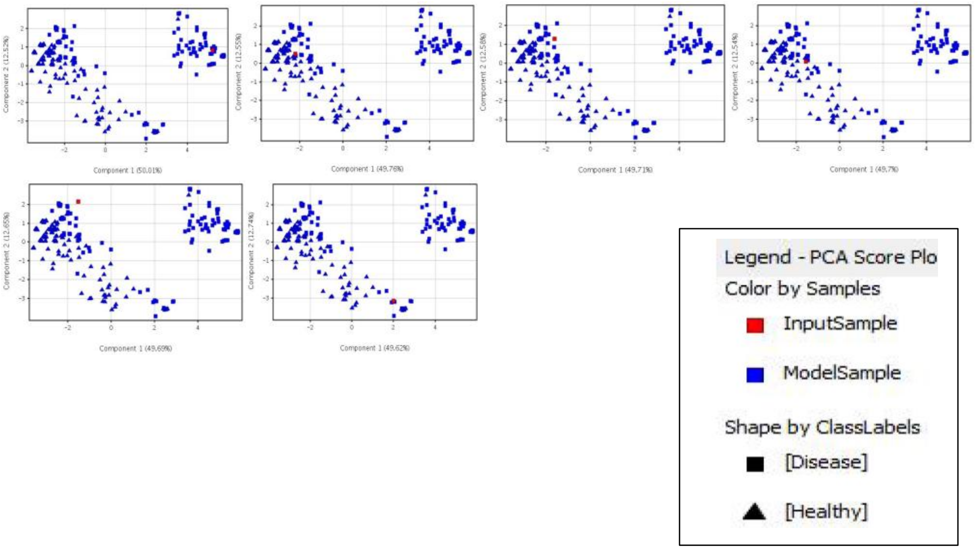
Legend - PCA Score Plo
Color by Samples
InputSample
ModelSample
Shape by ClassLabels
[Disease]
[Healthy]

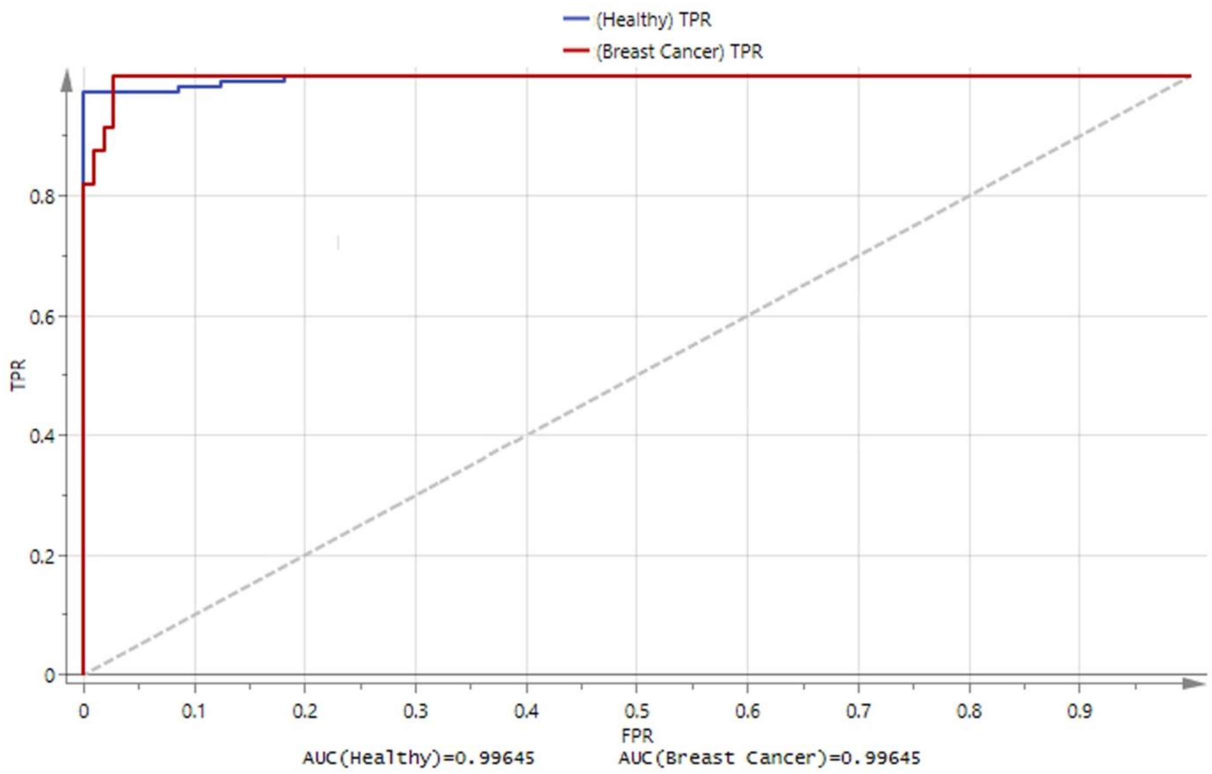

Figure S15.The ROC curves of prediction model for healthy and BC patients was found sufficiently good as the curve follows the left-hand border and the top border of the ROC space hence validating the accuracy of model.

Table S 10. List of metabolic pathways

Pathway	Total	Expected	Hits	Raw p	-log(p)	Holm adjust	FDR	Impact
Galactose metabolism	41	0.2	2	0.016	4.09	1	0.98	0.017
Starch and sucrose metabolism	50	0.25	2	0.02	3.71	1	0.98	0.059
Amino sugar and nucleotide sugar metabolism	88	0.44	2	0.068	2.68	1	1	0
Fructose and mannose metabolism	48	0.24	1	0.215	1.54	1	1	0.029
Fatty acid biosynthesis	49	0.24	1	0.219	1.52	1	1	0
Purine metabolism	92	0.46	1	0.374	0.98	1	1	0.004
